# A user guide for the online exploration and visualization of PCAWG data

**DOI:** 10.1038/s41467-020-16785-6

**Published:** 2020-07-07

**Authors:** Mary J. Goldman, Junjun Zhang, Nuno A. Fonseca, Isidro Cortés-Ciriano, Qian Xiang, Brian Craft, Elena Piñeiro-Yáñez, Brian D. O’Connor, Wojciech Bazant, Elisabet Barrera, Alfonso Muñoz-Pomer, Robert Petryszak, Anja Füllgrabe, Fatima Al-Shahrour, Maria Keays, David Haussler, John N. Weinstein, Wolfgang Huber, Alfonso Valencia, Peter J. Park, Irene Papatheodorou, Jingchun Zhu, Vincent Ferretti, Miguel Vazquez

**Affiliations:** 1 UC Santa Cruz Genomics Institute, Santa Cruz, CA 95064 USA; 2 Ontario Institute for Cancer Research, Toronto, ON M5G 0A3 Canada; 3 European Molecular Biology Laboratory, European Bioinformatics Institute, EMBL-EBI, Hinxton, CB10 1SD UK; 4 Department of Biomedical Informatics, Harvard Medical School, Boston, MA USA; 5 Centre for Molecular Science Informatics, Department of Chemistry, University of Cambridge, Lensfield Road, Cambridge, CB2 1EW UK; 6 Bioinformatics Unit, Spanish National Cancer Research Centre (CNIO), 28029 Madrid, Spain; 7 Data Sciences Platform, Broad Institute, Cambridge, MA USA; 8 Department of Bioinformatics and Computational Biology, UT MD Anderson Cancer Center, Houston, TX 77030 USA; 9 European Molecular Biology Laboratory, 69117 Heidelberg, Germany; 10 Barcelona Supercomputing Center (BSC), 08034 Barcelona, Spain; 11 ICREA, 08010 Barcelona, Spain; 12 CHU Sainte-Justine Research Center, Montreal, QC H3T 1C5 Canada; 13 Norwegian University of Science and Technology, Trondheim, Norway; 14 Present Address: European Molecular Biology Laboratory, European Bioinformatics Institute, Hinxton, UK

**Keywords:** Genomics, Cancer genomics, Cancer genomics

## Abstract

The Pan-Cancer Analysis of Whole Genomes (PCAWG) project generated a vast amount of whole-genome cancer sequencing resource data. Here, as part of the ICGC/TCGA Pan-Cancer Analysis of Whole Genomes (PCAWG) Consortium, which aggregated whole genome sequencing data from 2658 cancers across 38 tumor types, we provide a user’s guide to the five publicly available online data exploration and visualization tools introduced in the PCAWG marker paper. These tools are ICGC Data Portal, UCSC Xena, Chromothripsis Explorer, Expression Atlas, and PCAWG-Scout. We detail use cases and analyses for each tool, show how they incorporate outside resources from the larger genomics ecosystem, and demonstrate how the tools can be used together to understand the biology of cancers more deeply. Together, the tools enable researchers to query the complex genomic PCAWG data dynamically and integrate external information, enabling and enhancing interpretation.

## Abstract (web summary)

The Pan-Cancer Analysis of Whole Genomes project generated a vast array of data. In this article, the authors describe five different online resources to enable readers to explore and visualize the data.

## Introduction

The Pan-Cancer Analysis of Whole Genomes (PCAWG) Consortium aggregated whole-genome sequencing (WGS) data from 2658 cancers across 38 tumor types generated by the International Cancer Genome Consortium (ICGC) and The Cancer Genome Atlas (TCGA) projects. These sequencing data were re-analyzed with standardized, high-accuracy pipelines to align to the human genome (reference build hs37d5) and identify germline variants and somatically acquired mutations, as described in the PCAWG marker paper^1^. Here we provide a user guide to five tools introduced in the PCAWG marker paper: The ICGC Data Portal, UCSC Xena, Chromothripsis Explorer, Expression Atlas, and PCAWG-Scout. Each of them was created or extended to explore PCAWG data resources^1^. All of the tools aim to streamline analysis and visualization by pre-loading the PCAWG data so that users do not need to locate, curate, or manage the data and by making the tools accessible through a web interface. Each of these five tools also integrates other genomics datasets and tools that provide context and insight for interpretation of patterns in the PCAWG data helping this resource fully realize its potential. Some of the datasets and tools integrated include the UCSC Genome Browser^2^, Ensembl^3^, drug target compendia^4^, COSMIC^5^, and even large and complementary sequencing efforts such as GTEx^6^. Intuitive access to these additional tools and datasets is provided either by showing their data side by side or by providing context-dependent URL links.

**Fig. 1:**
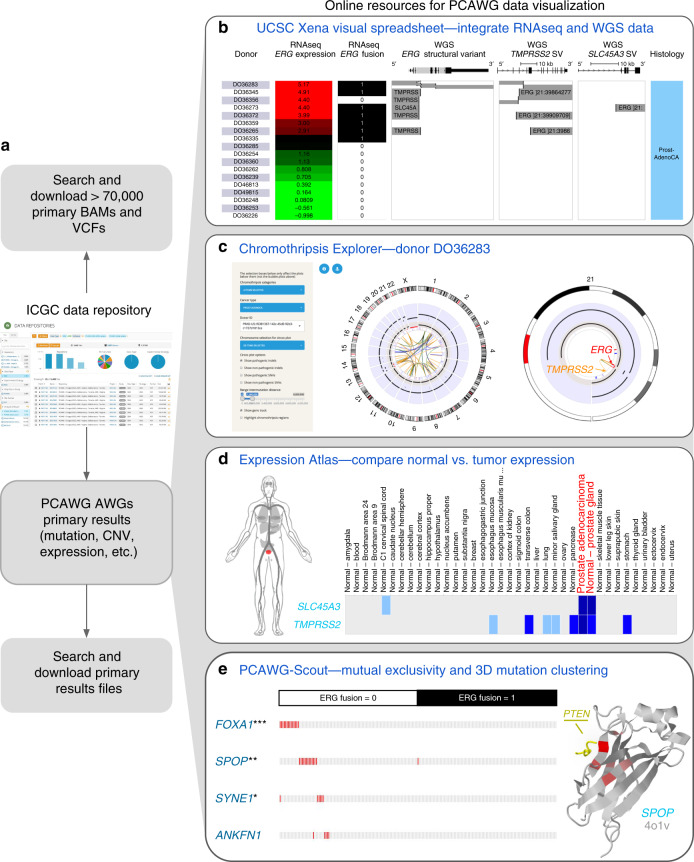
Synergy of the five tools. Instructions for reproducing the results shown are in Supplementary Note 2. **a** To obtain PCAWG BAMs, VCFs, and Analysis Working Group (AWG) files, the user selects the files desired, downloads a file manifest, and then downloads the actual data files (with authorization if needed) using the ICGC download tool. UCSC Xena, Chromothripsis Explorer, Expression Atlas, and PCAWG-Scout have each downloaded and processed the same primary analysis working group result files. **b** The UCSC Xena Visual Spreadsheet shows that the *ERG* fusion is present in 8 out of 18 PCAWG prostate adenocarcinoma samples (https://tinyurl.com/y78adbl5), as detected by the PCAWG RNA-seq and whole-genome sequencing data. Each row corresponds to a sample. Columns, starting at the left, correspond to histology, *ERG* gene expression, and *ERG* fusion based on RNA-seq data. The last three columns show structural variant calls using whole-genome DNA-seq data for *ERG*, *TMPRSS2*, and *SLC45A3*. **c** Chromothripsis Explorer provides an in-depth genome-wide view of copy number alterations and structural variations identified in the eight tumors with *ERG* fusion listed in **b**. Detailed information on total and minor copy number variations, as well as SVs, can be obtained by hovering over the elements within the Chromothripsis Explorer. Circos plot visualizations for the other 7 donors are given in Supplementary Fig. 4. **d** The Expression Atlas shows a heatmap of genes (rows) and tissue or disease type (columns). Here we show the expression of *TMPRSS2* and *SLC45A3* in healthy human tissue (top heatmap), as derived from our re-analysis of the GTEx dataset. The bottom heatmap shows expression in PCAWG data (https://tinyurl.com/y9fefymf). The human figure, called an anatomogram, shows the prostate tissue, highlighted in red. **e** PCAWG-Scout complements the above analysis by identifying recurrent mutational events in tumors without ERG fusion (fusion = 0). On the left is a mutation exclusivity analysis run by PCAWG-Scout (FDR-corrected Fisher’s exact test), which identifies *FOXA1* (***p < 0.0005), *SPOP* (**p < 0.005), *SYNE1* (*p < 0.05), as significantly associated with non-fusion tumors (https://tinyurl.com/qqudbkg). In the 3D protein structure of SPOP shown on the right, mutations are seen to cluster tightly around the region that overlaps with the interaction surface of *PTEN*. The portion of *PTEN* that interacts with *SPOP* is shown in yellow, along with the SPOP structure. Red indicates recurrent mutations in *SPOP*, with a brighter red indicating higher rate of recurrence.

The five resources in this paper each provide a different perspective and focus to the PCAWG data (Table 1). The ICGC Data Portal serves as the main entry point for accessing all PCAWG data and also enables exploration of PCAWG consensus simple somatic mutations, including point mutations and small indels, each by their frequencies, patterns of co-occurrence, mutual exclusivity, and functional associations. UCSC Xena integrates diverse types of genomic and phenotypic/clinical information at the sample level across the large number of samples, enabling rapid examination of patterns within and across data types. The Chromothripsis Explorer visualizes genome-wide mutational patterns, with a focus on complex genomic events, e.g., chromothripsis and kataegis. This is achieved through interactive Circos plots for each tumor with different tracks that correspond to allele-specific copy number variants, somatic structural variations, simple somatic mutations, indels, and clinical information. The Expression Atlas focuses on RNA-seq data, supporting queries in either a baseline context (e.g., finding genes that are expressed in prostate adenocarcinoma samples) or in a differential context (e.g., finding genes that are under- or over-expressed in prostate adenocarcinomas compared to adjacent normal prostate samples). PCAWG-Scout allows users to run their own analyses on-demand, including prediction of cancer-driver genes, differential gene expression, recurrent structural variations, survival, pathway enrichment, mutations as visualized on a protein structure, mutational signatures, and possible recommended therapies (based on the in-house PanDrugs resource; Supplementary Fig. 1). Each of the five tools offers different visualizations and analyses of the PCAWG data resource, each with its own strengths, and each enabling different insights into the data. When employed together, they provide the user with a deeper understanding of the cancer’s biology (Fig. 1). More information about the tools can be found at the PCAWG Landing Page (http://docs.icgc.org/pcawg).

**Table 1:** Search, visualization, analysis/integration, and download functionalities provided by each of the PCAWG data resources.

Functionality	ICGC Data Portal	UCSC Xena	Chromothripsis Explorer	Expression Atlas	PCAWG-Scout
***Search***					
Search by demographic data, specimen phenotype, molecular subtype	Y	Y			Y
Search by genes and/or variants	Y	Y		Y	Y
Search by genomic coordinates	Y	Y			
***Visualize***					
Visualize multiple types of data together		Y	Y	Y	Y
Visualize coding variants	Y	Y	Y		Y
Visualize non-coding variants	Y	Y	Y		
Visualize structural variants		Y	Y		Y
Visualize mutational signatures and predicted drivers					Y
Visualize genome-wide profiles, including LOH, in Circos plots			Y		
Visualize tissue expression on a human figure				Y	
Visualize gene co-expression		Y		Y	
Visualize pathways, therapeutic associations	Y				Y
Visualize summary of BAMs/VCFs	Y				
***Analysis***					
Kaplan–Meier analysis with statistics	Y	Y			Y
Gene set/pathway enrichment analysis	Y			Y	Y
View non-identifiable analysis results of protected data	Y		Y	Y	Y
Discover differentially or co-expressed genes, mutually exclusive genomic events					Y
Annotations from other resources	Y	Y		Y	Y
***Download***					
Programmatic data download	Y	Y		Y	Y
Download BAMs, VCFs, primary files	Y				
Download secondary processed data	Y	Y		Y	Y

## Results

### ICGC Data Portal and a use case

As a main entry point, the ICGC Data Portal (https://dcc.icgc.org, Zhang^7^) provides an intuitive graphical interface for browsing, searching, and visualizing PCAWG datasets (Fig. 1a). Uniformly aligned sequencing BAM files and variant calling VCF files, although physically residing in multiple repositories globally, can be centrally searched via the ICGC Data portal (https://icgc.org/ZEA). Users can readily find specific datasets of interest with a few mouse clicks using various facet terms to narrow their search. Other downstream analysis results generated by PCAWG working groups are available at https://dcc.icgc.org/releases/PCAWG. Close to 23 million open access PCAWG consensus simple somatic mutations have been annotated with consequences for protein structure, affected pathways, targeting cancer drugs, gene ontology terms, and clinical parameters. The portal’s Advanced Search (https://icgc.org/ZzP) tool allows users to perform complex queries, for example, to retrieve the most frequently mutated targets of drugs in stage 2 liver cancers (https://icgc.org/ZHe). Analytic tools, including access to a Jupyter Notebook sandbox for advanced users, support exploration of potential associations between molecular abnormalities and phenotypic observations such as donor survival (https://dcc.icgc.org/analysis). The ICGC Data Portal publicly displays non-identifiable, aggregated analysis results from protected data.

The ICGC Data Portal is best for users who are seeking to download PCAWG data for their own analyses. It also includes the richest resources and functionality for users interested in single-nucleotide variants (SNVs), including patterns of co-occurrence, mutual exclusivity, and functional associations. Figure 1a shows an example use case that demonstrates how bioinformaticians and other tool creators can download results from the portal and then run their own analyses or offer their own visualizations of the data.

### UCSC Xena and a use case

**Fig. 2:**
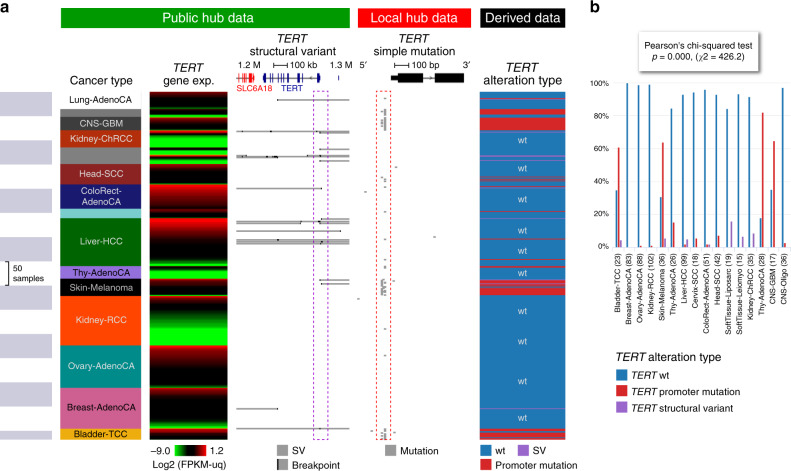
UCSC Xena views of *TERT* across cancer types. **a** Visual spreadsheet view of *TERT* multi-omics data across PCAWG cancer types. Data from the PCAWG public hub are under the green section, and protected data from the user’s local Xena hub are under the red section. The public and private datasets are integrated in the browser, keeping the private data protected. Many of the cancer types show *TERT* alterations, either as simple somatic mutations in the promoter region (as seen in the pileup highlighted in the red box) or as structural variants (as seen in the breakpoint pileup upstream of *TERT* highlighted in the purple box). Only cancer types that have a *TERT* alteration are displayed (*n* = 718 samples). The last column, dynamically generated in the browser, shows which samples have promoter mutations, which have structural variants, and which have none. No sample is observed to have both promoter mutations and structural variants; hence, the two types of alterations are mutually exclusive. **b** Distribution of different types of *TERT* alterations across cancer types, as shown in Xena chart view. Xena automatically runs the appropriate statistical test for every chart; in this case, Xena calculated that the difference in distributions across cancer types is statistically significant.

UCSC Xena’s (https://pcawg.xenahubs.net) adaptable visualizations, fast performance, and flexible data format make the full power of the PCAWG resource available to all researchers^8^. It displays data mapped to coding and non-coding regions of the genome, including introns, promoters, enhancers, and intergenic regions. Xena can display tens of thousands of data points on thousands of samples, all within seconds. The Xena Browser excels at integrating the diverse datasets generated by the PCAWG Consortium using the Xena Visual Spreadsheet, which enables users to view multiple types of data side by side (Fig. 1b). In addition to the Visual Spreadsheet, Xena offers survival analyses, the ability to compare and contrast dynamically built subgroups, statistical tests such as analysis of variance, and URLs to live visualizations for sharing with collaborators or others. Xena’s hub-browser architecture enables users to view the protected consensus simple somatic mutations, including non-coding mutations, by loading the dataset into a user’s local private Xena hub (Fig. 2, Supplementary Fig. 2). The Xena Browser seamlessly integrates data from multiple hubs, allowing users who have access to the protected mutation data to visualize it in conjunction with other PCAWG data publicly available on the PCAWG Xena Hub (https://pcawg.xenahubs.net).

UCSC Xena is best for integrating diverse PCAWG data types, including simple mutations, gene expression levels, and gene fusions, as well as less common types such as alternative splicing^1^ events, promoter usage, and mutational signature scores, all from the same set of samples (Supplementary Note 1). It also provides a mechanism for viewing protected non-coding SNVs either separately or in conjunction with other PCAWG data. Figure 2 shows an example use case, exploring alterations in the *TERT* gene. Both public data (structural variants (SVs)) and private data (SNVs) on the *TERT* gene are shown. The data are integrated in the browser, keeping private data protected. Even though the data are distributed across multiple hubs with different access controls, they appear to the user to come from a unified dataset, allowing easy visualization and data integration. Figure 2 shows alterations by SNV and alterations by larger structural variation that are mutually exclusive. We also see that there are significant differences in the type of alteration in different cancer types (chi-square, one-sided, *F* = 426.2, *p* < 0.001).

### Chromothripsis Explorer and a use case

Chromothripsis refers to a mutational process characterized by massive de novo rearrangements that affect one or multiple chromosomes^9^. The whole-genome dataset assembled by PCAWG permitted us to characterize chromothripsis patterns on a large scale at single-base resolution across >30 cancer types^10^. Although chromothripsis is generally identified by statistical metrics^11^, visual inspection still remains essential to dismiss false-positive cases generated by other mechanisms of genome instability^10,12^. The Chromothripsis Explorer (http://compbio.med.harvard.edu/chromothripsis/) is an open source R Shiny application that visualizes chromothripsis patterns detected using WGS data^1,10^.

**Fig. 3:**
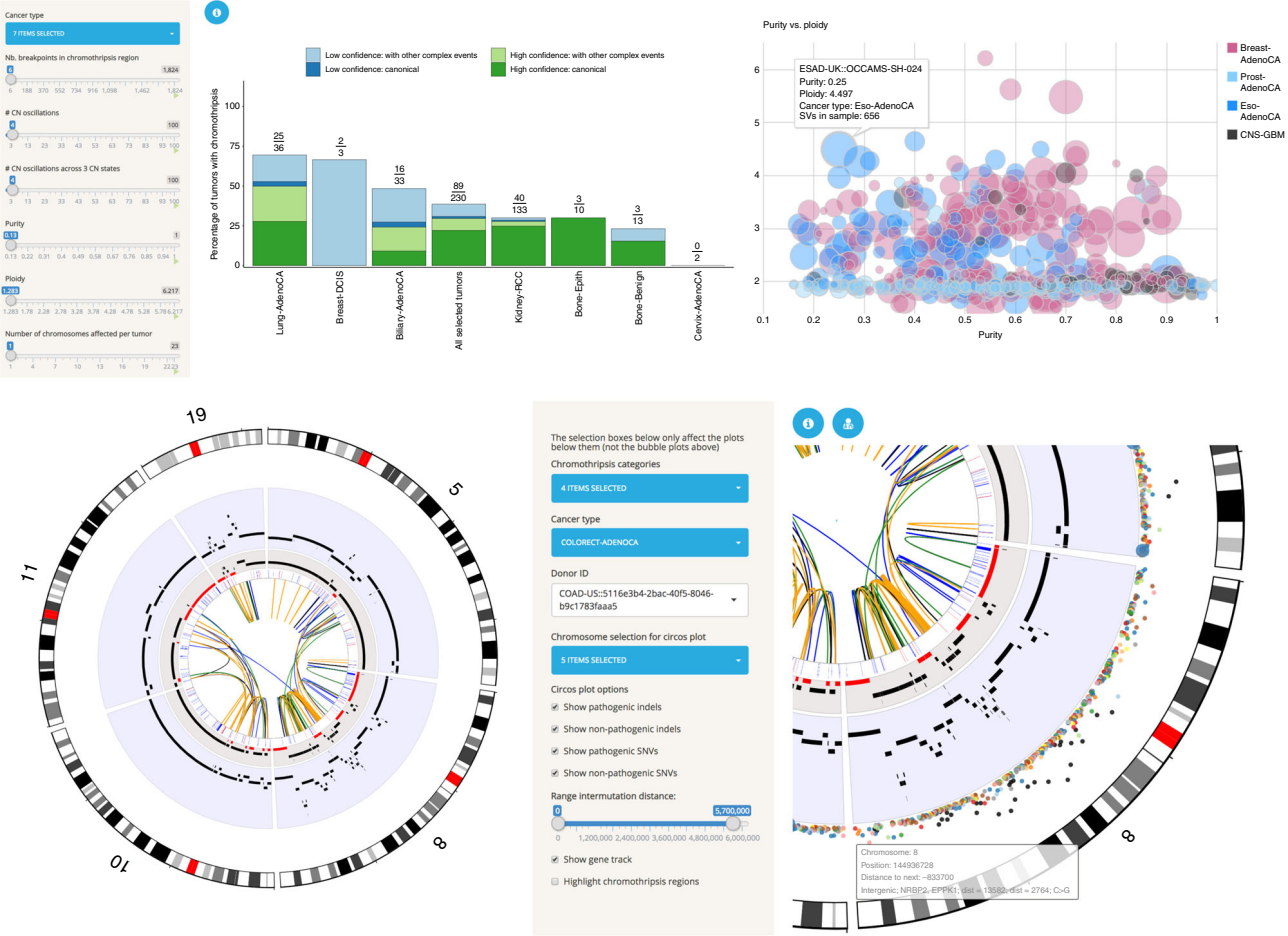
Functionalities of the Chromothripsis Explorer. **a** Interactive bar plot for visualization of chromothripsis rates for selected cancer types. The left-hand side panel shows variables used for detection of chromothripsis patterns (e.g., number of copy number oscillations; Cortés-Ciriano^10^). The user can modify the values of those variables to explore chromothripsis rates as a function of stringency criteria. The right-hand panel shows additional functionalities for exploring the relationship between purity and ploidy for tumors of selected cancer types. **b** Visualization of complex rearrangements involving five chromosomes in a ColoRect-AdenoCA donor (ICGC ID: DO9034). The right-hand panel shows a zoomed view of chromosome 8 that illustrates the tracks available in the Circos plots. From the outer to the inner ring, the tracks correspond to hg19 cytobands, SNVs (colored according to the mutation type and distributed according to the inter-mutation distance), total copy number (over a blue background), minor copy number (LOH regions, with a minor copy number equal to 0 depicted in red), gene track, and SVs. Further information about the tracks can be accessed by clicking on the blue information circle located above the Circos plot.

The Chromothripsis Explorer provides tools for exploration of chromothripsis frequencies and patterns across tumor types (Fig. 3a). Specifically, it provides interactive Circos plots^13^ for each tumor, allowing researchers to explore large-scale alterations such as chromosome arm deletions and complex mutational patterns such as chromothripsis and chromoplexy (Fig. 3b). Each Circos plot is divided into seven tracks that display, from outer to inner rings: (i) hg19 cytobands; (ii) inter-mutation distance and location for pathogenic (i.e., non-synonymous, stop-gain, and stop-loss) and nonpathogenic SNVs, as well as frame-shift and in-frame indels; (iii) chromothripsis regions; (iv) total copy number; (v) minor copy number profiles, defined as the least amplified allele, to visualize loss of heterozygosity (LOH) regions; (vi) gene annotation track, and (vii) structural variations displayed according to read orientations at the breakpoints (duplication-like SVs in blue, deletion-like SVs in orange, head-to-head inversions in black, and tail-to-tail inversions in green). By hovering over a Circos plot, the user can obtain information about a mutation of interest at single-base resolution and also see gene annotations and functional effect predictions. In addition to the genomic data, clinical and histo-pathological information are provided for all tumors in the form of customizable tables that enable the user to map tumor identifiers across cancer projects (e.g., TCGA to ICGC IDs; Fig. 3b).

The Chromothripsis Explorer is best for users who are looking for a global picture of somatic alterations in a tumor (e.g., large-scale aneuploidies or translocations). It also provides visualizations of the point mutations, as well as small insertions and deletions, on a genome-wide scale. A representative use case for Chromothripsis Explorer is the exploration of complex rearrangements in one or more human cancers, as shown in Fig. 3b for ColoRect-AdenoCA tumor ICGC ID: DO9034. By selecting the chromosomes that harbor massive rearrangements, in this case chromosomes 5, 8, 10, 11, and 19, the user can investigate the consequences of complex rearrangements such as LOH across chromosome 8 and copy number amplifications in multiple locations.

### Expression Atlas and a use case

**Fig. 4:**
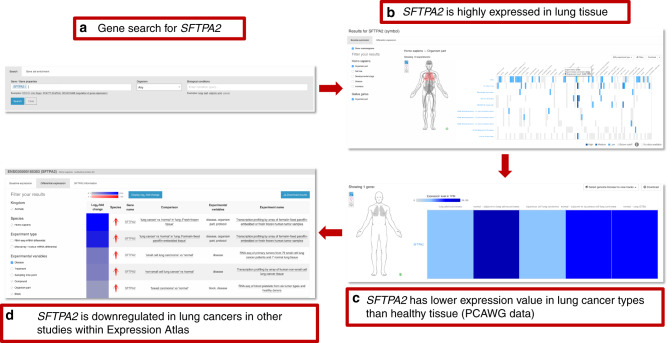
Example of a gene search in Expression Atlas. **a** Searching for experiments in which *SFTPA2* is expressed or differentially expressed. **b** Viewing expression of *SFTPA2* in different tissues and across all baseline experiments. *SFTPA2* shows consistently high expression in the lung. **c** Looking for the same gene in the PCAWG study using Expression Atlas. Expression is low in lung cancers (adenocarcinoma and squamous cell) but high in the corresponding adjacent normal tissue samples. Expression in normal lung is also high in GTEx. **d** Finally, the downregulation of *SFTPA2* is confirmed in further differential assay studies of lung cancer presented in Expression Atlas.

Expression Atlas (https://www.ebi.ac.uk/gxa/experiments/E-MTAB-5200/, Petryszak^14^) is an added-value database and web service that enables the user to assess gene expression in different tissues, cell types, diseases, and developmental stages. It collects, annotates, re-analyses, and displays gene, transcript, and protein expression data. It supports two types of study design: baseline and differential. Baseline studies involve quantitation of genes by tissue type, developmental stage, cell line, cancer type, or other factors. Differential studies perform expression comparisons between different samples, for example, disease vs. healthy tissue (Fig. 4). In addition to the PCAWG datasets, selected expression studies from archives such as ArrayExpress, GEO (Gene Expression Omnibus) and ENA (European Nucleotide Archive) also underwent further curation and processing. Data curation is semi-automated and involves identifying the experimental factors, such as diseases or perturbations, annotating metadata with Experimental Factor Ontology (EFO) terms, and describing the experimental comparisons for further processing. Currently, Expression Atlas provides results from >3500 experiments that include about 120,000 assays from >60 different organisms. The datasets cover >100 cell types from the Cell Ontology and >700 diseases represented in the EFO.

Expression Atlas includes differential studies on human diseases in humans and animal models as well as large baseline studies on human subjects or cell lines, including GTEx, CCLE, ENCODE, BLUEPRINT, and HipSci. Analyses of bulk or single-cell RNA-seq datasets are performed using our open source pipeline iRAP^15^. Expression Atlas can be searched by gene, gene set, or experimental condition (Fig. 4a). Gene, transcript, and protein expression across different conditions are displayed through heatmaps and boxplots (Fig. 4b). Annotation of datasets with EFO terms enables nested searching across related tissues, diseases, and other conditions modeled within EFO. For example, a search for “cancer” will produce results for all different types of cancer, including “leukemia.” PCAWG datasets can be viewed and queried within their *study pages* or they can be viewed alongside other studies within Expression Atlas, returned as matches to gene or condition queries from the home page.

Expression Atlas is best for users who are interested in viewing how PCAWG gene expression data compare with those from other sources, especially normal tissues in GTEx. It also provides the ability to see gene expression on an anatomical figure, making it easy to visualize patterns of expression across the body. An example use case in Fig. 4 shows a typical gene search, in this case for gene *SFTPA2*, to identify in which tissues it is expressed and under what conditions its expression changes. The results of the query show high expression in lung tissue across different baseline expression studies available through Expression Atlas. Focusing on the PCAWG datasets, we see that expression is low in lung cancers (adenocarcinoma and squamous cell carcinoma), whereas it is highly expressed in the corresponding adjacent normal tissues. It is also highly expressed in lung samples from GTEx. Finally, through the panel of available differential studies (bulk RNA-Seq or microarray), the user can confirm from additional studies in Expression Atlas that *SFTPA2* is downregulated in lung cancers.

### PCAWG-Scout and a use case

**Fig. 5:**
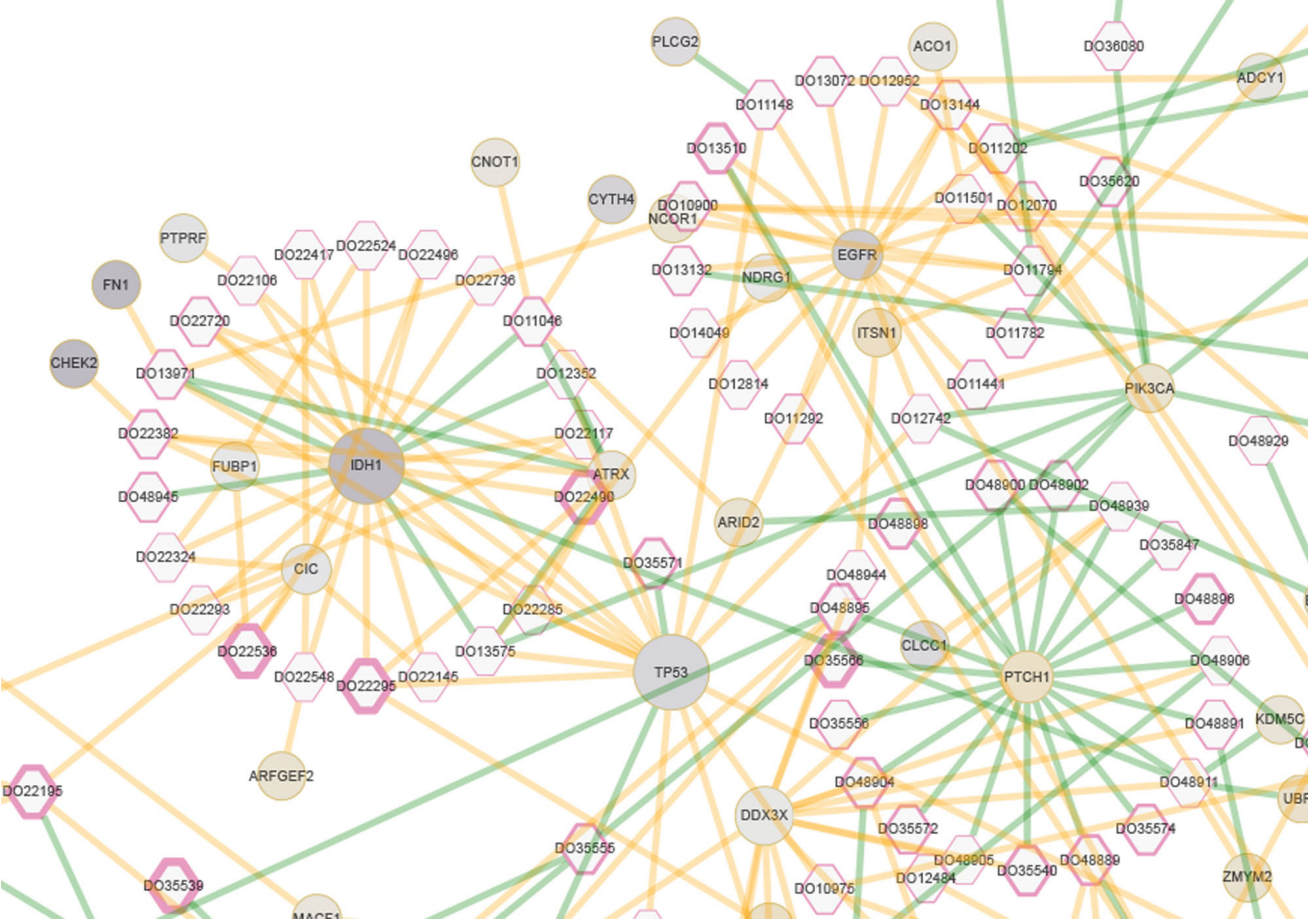
PCAWG Central Nervous System meta-cohort donor-driver events in PCAWG-Scout. This Cytoscape-based visualization, available from the Study report, shows donors as hexagons and genes as circles. The PCAWG (Central Nervous System) CNS meta-cohort consists of the samples from cohorts identified by the codes CNS-GBM, CNS-Medullo, CNS-Oligo, CNS-PiloAstro. Edges represent driver events that either were validated or were only predicted by the PCAWG Drivers Working Group; validated driver events are colored green and predicted driver events are colored orange. Most of the driver events for *IDH1* are shown as only predicted, whereas all of those for *PTCH1* have been validated. The hexagon border thickness for each donor corresponds to the reported survival time. The size of each gene circle is proportional to the extent to which mutations found in the cohort for that gene have damage scores (MetaLR_score from DbNSFP v3.2a) higher than the scores for all possible SNVs that can happen over that gene (one-sided *t* test; the sample size varies from gene to gene). Genes *IDH1*, *TP53*, and *DDX3X* stand out as being more damaged than expected by chance. The circle color for each gene corresponds to its differential expression as calculated by PCAWG-Scout (FDR-corrected two-sided *t* statistic) when comparing *IDH1*-mutant samples with *IDH1*-wild type tumor samples; purple, light gray, and gold denote underexpression, no significant differential, and overexpression, respectively. The upper and lower bounds of the coloring gradient are defined on the basis of the entire genome, not just for the genes represented in the graph. *IDH1* is among the most under-expressed, along with *FN1* and *CHEK2*. *FN1* and *CHEK2* have driver events that co-occur in some samples with those for *IDH1*. *PTCH1* is overexpressed in *IDH1* mutants. Graphical esthetics of node border width, node size, node color, and edge color are configurable interactively. Details of the analysis and instructions for reproducing this visualization are available in Supplementary Note 3.

As opposed to offering only a limited and predefined list of analyses, PCAWG-Scout (http://pcawgscout.bsc.es/) offers a variety of on-demand analysis functionalities. The analyses enable researchers to explore and visualize the data, form a hypothesis, run the relevant analysis, and immediately explore and visualize the results, giving rise to an analysis loop that drives discovery. The analyses are performed on data from the PCAWG main data release (available in the ICGC data repository) and on results from the PCAWG working groups. Results from the working groups include driver calls for different cohorts and for individual samples, mutation clonality assignments, and mutational signatures, all of which are integrated into different sections of the PCAWG-Scout reports, tables, and interactive visualization graphics. PCAWG-Scout generates a set of visualizations and analyses, called a report, on any number of cohorts, samples, or genes. Reports include descriptions, statistics, plots, interactive three-dimensional (3D) protein representations, and network graphs (Fig. 5). The reports also offer additional, optional analyses, including enrichment analysis of gene lists, driver predictions over cohorts, survival analysis for lists of samples, and potential recommended therapies for individual donors (Supplementary Fig. 3). PCAWG-Scout uses a plugin approach that makes it easy for the user to customize reports or perform new types of analyses. Data and results are exported in interoperable formats to help integrate PCAWG-Scout with other software packages.

PCAWG-Scout is best for users who are looking for a web interface to run analyses on PCAWG data (e.g., differential gene expression or gene set enrichment). It also offers 3D mutation views for coding SNVs and INDELs. The potential to explore PCAWG data in PCAWG-Scout is illustrated in Fig. 5, which shows a network visualization tool that was configured from the web interface with parameters gathered through analyses run within the tool itself. The tool offers the user a bird’s eye view of a number of important facets of the biology, in the case of Fig. 5, of central nervous system tumors. For instance, *IDH1*, *TP53*, and *DDX3X* stand out as genes in which mutations are more damaging than expected. Plots such as these can help the user identify patterns such as mutual exclusivity and clinical prognosis, as well as highlight the ways in which gene function can be deregulated, for example, by mutation or alteration of gene expression.

### Synergy of the different tools

Combining the strengths of the different tools can provide a deeper understanding of tumor biology. That synergy is illustrated by considering a common driver event in prostate cancer: fusion of the oncogene *ERG*^16,17^ (Fig. 1). Xena’s Visual Spreadsheet enables the user to look across all 18 PCAWG prostate samples with both WGS and RNA-seq data, showing that 8 of the samples harbor an *ERG* fusion. These samples also show *ERG* overexpression (Fig. 1b). A view of the PCAWG SV data shows that, across all samples, the fusion breakpoints are located at the *ERG* transcription start site, leaving the *ERG*-coding region intact and fusing it to the promoter region of *TMPRSS2* or *SLC45A3* (Fig. 1b). In addition, the figure shows that fusions detected by RNA-seq and WGS are not always consistent; one fusion detected by a consensus of RNA-based detectors is missed in the WGS calls, and the converse is also seen. This example shows that an integrated visualization across multiple data types and algorithms can provide a more accurate picture of a genomic event.

The Chromothripsis Explorer adds a more in-depth view of the CNV and SV alterations in the eight tumors with *ERG* fusions. It shows that alterations in those eight tumors vary widely (Fig. 1c, Supplementary Fig. 4). Whereas donors DO36372, DO36359, DO36265, and DO36335 have quiescent genomes with few SVs, DO36356 and DO36283 show more complex karyotypes. For example, in DO38283, chromosome 21 harbors multiple SVs that link it with chromosomes 2, 9, 13, and 21 (right). A closer look at the intrachromosomal SVs in chromosome 21 (left) reveals an oncogenic fusion generated by a deletion at chr21:39,988,805–40,578,907.

The Expression Atlas adds the observation that expression levels of *TMPRSS2* and *SLC45A3* vary across tissue and tumor types but that both *TMPRSS2* and *SLC45A3* are highly expressed in normal prostate tissues and prostate tumors, as shown in the Expression Atlas Baseline Expression Widget (Fig. 1d). Combined analysis of the PCAWG and GTEx datasets leads to the hypothesis that a subset of prostate cancers, through genome rearrangement, hijack the promoters of androgen-responsive genes to increase *ERG* expression, resulting in an androgen-dependent overexpression of *ERG*.

PCAWG-Scout adds further information by illuminating genomic events in the prostate samples that do not show ERG fusions. Although *ERG* fusions are frequent, 46% (89 out of 195) of the PCAWG prostate tumors do not show them (Supplementary Fig. 5). In fact, we can see using PCAWG-Scout’s mutual exclusivity analysis that simple mutations in *FOXA1*, *SPOP*, and *SYNE1* are significantly associated with non-fusion tumors (Fig. 1e). Furthermore, in PCAWG-Scout’s 3D protein structure view, the mutations in *SPOP* cluster tightly around the interaction interface for *PTEN* (Fig. 1e), suggesting that those mutations may lead to altered *SPOP* protein function.

The use case in this section highlights some of the strengths of each individual tool and also demonstrates how the tools can be used synergistically to gain a fuller understanding of a genomic event, in this case *ERG* fusions in prostate cancer. In this example, we started with UCSC Xena, but the user can start with any of the five tools and then use others to investigate further.

## Discussion

The data generated by the PCAWG consortium provide a valuable resource for understanding complex cancer biology. Here we have described five tools that aim to put that resource into the hands of all researchers and also incorporate outside genomic data resources. Those tools, the ICGC Data Portal, UCSC Xena, Chromothripsis Explorer, Expression Atlas, and PCAWG-Scout, are all available at The PCAWG Data Portals and Visualization Page (http://docs.icgc.org/pcawg). Visualization of patterns within the PCAWG data is challenging because of the relatively large number of whole genomes studied, the large size of each dataset at the sequence level, and the difficulty of viewing all intergenic and intronic regions explicitly at either the sequence or gene level. Those factors impose high-performance requirements for interactive tools, especially those on the web. Adding to the high-performance requirements is the challenge of visualizing the wide array of data types derived from the high-quality genomic information provided by whole-genome data, including point mutations, gene fusions, promoter usage, and SVs. Many visualization tools, especially those for users without extensive computational training, are currently limited to coding regions and more typical genomic datasets such as those on SNVs or CNVs; they are not able to take full advantage of the depth and complexity of information made available by the PCAWG consortium. Each of the tools presented here was either created or extended in the context of the PCAWG project to address those whole-genome visualization challenges.

Nevertheless, we expect that other tools will be developed to address the visualization challenges associated with the whole-genome PCAWG data. In fact, another group in the PCAWG consortium has created an online tool to explore the panorama of driver mutations in PCAWG tumors. The tool can be found via Gitools interactive heatmaps^18^ (http://www.gitools.org/pcawg). We hope that further visualization and analysis tool development will be facilitated by the open-source code for the tools described here (Supplementary Table 1), as well as by embeddable javascript modules for some tools’ functionalities (Supplementary Table 2).

### Reporting summary

Further information on research design is available in the Nature Research Reporting Summary linked to this article.

## Supplementary information

Supplementary Information

Reporting Summary

## Data Availability

Somatic and germline variant calls, mutational signatures, subclonal reconstructions, transcript abundance, splice calls, and other core data generated by the ICGC/TCGA Pan-cancer Analysis of Whole Genomes Consortium is described here^1^ and available for download at https://dcc.icgc.org/releases/PCAWG. Additional information on accessing the data, including raw read files, can be found at https://docs.icgc.org/pcawg/data/. In accordance with the data access policies of the ICGC and TCGA projects, most molecular, clinical, and specimen data are in an open tier, which does not require access approval. To access genetically sensitive information, such as germline alleles and underlying sequencing data, researchers will need to apply to the TCGA Data Access Committee (DAC) via dbGaP (https://dbgap.ncbi.nlm.nih.gov/aa/wga.cgi?page=login) for access to the TCGA portion of the dataset and to the ICGC Data Access Compliance Office (DACO; http://icgc.org/daco) for the ICGC portion. In addition, to access somatic single-nucleotide variants derived from TCGA donors, researchers will also need to obtain dbGaP authorization. Derived datasets within each tool can be found in Supplementary Table 3. The source data underlying Figs. 1–5, excepting the controlled data, are provided as a Source data file. Corresponding authors for respective tools: ICGC Data Portal, J. Zhang, junjun.zhang@oicr.on.ca; UCSC Xena, J. Zhu, jzhu@soe.ucsc.edu; Chromothripsis Explorer, P.J.P., peter_park@hms.harvard.edu; Expression Atlas, I.P., irenep@ebi.ac.uk; PCAWG-Scout, M.V., miguel.vazquez.g@bsc.es.

## Code availability

The core computational pipelines used by the PCAWG Consortium for alignment, quality control, and variant calling are available to the public at https://dockstore.org/search?search=pcawg under the GNU General Public License v3.0, which allows for reuse and distribution. The code for all tools in this paper are open source and publicly available. Code for the ICGC Data Portal is available at https://github.com/icgc-dcc/dcc-portal. Code for the UCSC Xena Browser is available at https://github.com/ucscXena/ucsc-xena-client. Code for the Chromothripsis Explorer is available at https://github.com/parklab/ShatterSeek. Code for the Expression Atlas is at https://github.com/gxa/atlas. Code for PCAWG-Scout is at http://mikisvaz.github.io/rbbt/, https://github.com/Rbbt-Workflows, and https://github.com/Rbbt-Apps/PCAWGScout.
