# Supplementary material for: A user guide for the online exploration and visualization of PCAWG data
Source: Nat Commun. 2020 Jul 7;11:3400. doi: 10.1038/s41467-020-16785-6 (PMC7340791; doi:10.1038/s41467-020-16785-6)
Supplement: Supplementary file 1 — Supplementary Information [file 41467_2020_16785_MOESM1_ESM.pdf]

Supplementary Note 1. PCAWG data wrangling carried out in UCSC Xena.

PCAWG analysis working group primary results files (listed in the Data Availability Section) were downloaded and wrangled into two Xena cohorts: (i) the ‘PCAWG donor-centric’ cohort, in which all datasets use ICGC Donor IDs, such as DO217962 and (ii) the ‘PCAWG specimen-centric’ cohort, in which all datasets use ICGC Specimen IDs, such as SP117136.

Several steps were taken to wrangle all genomic and phenotypic datasets into Xena. For genomic datasets, we mapped data from aliquot IDs to donor IDs for the donor-centric cohort, and to specimen IDs for the specimen-centric cohort. Specific to the donor-centric cohort, data from normal specimens were removed, so the data represent only tumor profiles. When multiple specimens were available for the same donor, an average was taken and assigned to the donor.

For phenotype data, we mapped the specimen histology classifications back to the donor IDs after data from normal specimens were removed. We propagated donor clinical data to all specimen IDs belonging to the donor.

We extracted coding mutations from the consensus simple somatic mutation datasets and made the resulting dataset available on the open-access PCAWG hub (Consensus SNVs and indels - coding). The protected whole-genome consensus dataset containing simple somatic mutations was downloaded and wrangled into Xena-ready format. The resulting Xena-ready file was uploaded to GNOS (https://gtrepo-osdc-tcga.annailabs.com/cghub/metadata/analysisFull/86a1f73a-fabf-4abe-a597-a47d84a8c980), in which the data remains under controlled access.

Supplementary Note 2. Recreating the panels in Figure 1.

ICGC Data Repository (1a)
Figure 1a shows a view of the PCAWG data files in the ICGC Data Repository. It can be accessed through
https://dcc.icgc.org/repositories?filters=%7B%22file%22:%7B%22study%22:%7B%22is%22:%5B%22PCAWG%22%5D%7D%7D%7D&files=%7B%22from%22:1%7D
Shortened url: https://tinyurl.com/yburnde5

UCSC Xena Visual Spreadsheet (1b)
Figure 1b shows a view of the *ERG* fusion based on RNAseq and DNAseq data. It can be accessed through
https://xenabrowser.net/heatmap/?bookmark=29d8db43ebacda4f54e666d7666cf164
Shortened url: https://tinyurl.com/y78adbl5

Chromothripsis Explorer (1c)
Figure 1c shows a Circos plot visualization of a donor (DO38283) for whom fusion of the oncogene *ERG* was detected. To reproduce Figure 1c; (i) visit

http://compbio.med.harvard.edu/chromothripsis/; (ii) click on the blue box containing the text 'CLICK HERE TO EXPAND' below the panel 'Find cases of interest quickly!'; (iii) type the ICGC donor ID for the tumor (e.g., DO38283 for the first result listed in Figure 1b) in the search bar below the column 'ICGC donor ID'. The table shows associated information for the donor, including the donor_unique_id (e.g., PRAD-US::f6381367-142c-45d0-92b3-c1727d1813ce); (iv) go to the panel 'Interactive circos plots reporting SNVs, indels, total CNV, minor CN (LOH), and SV calls' and select (e.g., 'PROST-ADENOCA') in the dropdown menu 'Cancer type' located on the left-hand side menu. Next, select the row corresponding to the donor of interest (e.g., PRAD-US::f6381367-142c-45d0-92b3-c1727d1813ce) in the dropdown menu 'Donor ID'; (v) finally, select the number of chromosomes to display in the Circos plot using the dropdown menu 'Chromosome selection for circos plot'.

Expression Atlas (1d)

The query in Figure 1d, showing PCAWG data for prostate adenocarcinoma together with adjacent normal tissue and normal prostate gland tissue from GTEx can be accessed through

https://www.ebi.ac.uk/gxa/experiments/E-MTAB-5200/Results?specific=true&geneQuery=%255B%257B%2522value%2522%253A%2522Erg%2522%252C%2522category%2522%253A%2522symbol%2522%257D%252C%257B%2522value%2522%253A%2522tmprss2%2522%252C%2522category%2522%253A%2522symbol%2522%257D%252C%257B%2522value%2522%253A%2522SLC45A3%2522%252C%2522category%2522%253A%2522symbol%2522%257D%255D&filterFactors=%257B%2522DISEASE%2522%253A%255B%2522normal%2520-%2520blood%2520%28GTEx%29%2522%252C%2522normal%2522%252C%2522normal%2520-%2520amygdala%2520%28GTEx%29%2522%252C%2522normal%2520-%2520Brodmann%2520%281909%29%2520area%252024%2520%28GTEx%29%2522%252C%2522normal%2520-%2520Brodmann%2520%281909%29%2520area%25209%2520%28GTEx%29%2522%252C%2522normal%2520-%2520C1%2520segment%2520of%2520cervical%2520spinal%2520cord%2520%28GTEx%29%2522%252C%2522normal%2520-%2520caudate%2520nucleus%2520%28GTEx%29%2522%252C%2522normal%2520-%2520cerebellar%2520hemisphere%2520%28GTEx%29%2522%252C%2522normal%2520-%2520cerebellum%2520%28GTEx%29%2522%252C%2522normal%2520-%2520cerebral%2520cortex%2520%28GTEx%29%2522%252C%2522normal%2520-%2520hippocampus%2520proper%2520%28GTEx%29%2522%252C%2522normal%2520-%2520hypothalamus%2520%28GTEx%29%2522%252C%2522normal%2520-%2520nucleus%2520accumbens%2520%28GTEx%29%2522%252C%2522normal%2520-%2520putamen%2520%28GTEx%29%2522%252C%2522normal%2520-%2520substantia%2520nigra%2520%28GTEx%29%2522%252C%2522normal%2520-%2520breast%2520%28GTEx%29%2522%252C%2522normal%2520-%2520esophagogastric%2520junction%2520%28GTEx%29%2522%252C%2522normal%2520-%2520esophagus%2520mucosa%2520%28GTEx%29%2522%252C%2522normal%2520-%2520esophagus%2520muscularis%2520mucosa%2520%28GTEx%29%2522%252C%2522n

ormal%2520-
%2520cortex%2520of%2520kidney%2520%28GTEx%29%2522%252C%2522normal%2520-
%2520sigmoid%2520colon%2520%28GTEx%29%2522%252C%2522normal%2520-
%2520transverse%2520colon%2520%28GTEx%29%2522%252C%2522normal%2520-
%2520liver%2520%28GTEx%29%2522%252C%2522normal%2520-
%2520lung%2520%28GTEx%29%2522%252C%2522normal%2520-
%2520minor%2520salivary%2520gland%2520%28GTEx%29%2522%252C%2522normal%252
0-%2520ovary%2520%28GTEx%29%2522%252C%2522normal%2520-
%2520pancreas%2520%28GTEx%29%2522%252C%2522prostate%2520adenocarcinoma%25
22%252C%2522normal%2520-
%2520prostate%2520gland%2520%28GTEx%29%2522%252C%2522normal%2520-
%2520skeletal%2520muscle%2520tissue%2520%28GTEx%29%2522%252C%2522normal%2
520-%2520lower%2520leg%2520skin%2520%28GTEx%29%2522%252C%2522normal%2520-
%2520suprapubic%2520skin%2520%28GTEx%29%2522%252C%2522normal%2520-
%2520stomach%2520%28GTEx%29%2522%252C%2522normal%2520-
%2520thyroid%2520gland%2520%28GTEx%29%2522%252C%2522normal%2520-
%2520urinary%2520bladder%2520%28GTEx%29%2522%252C%2522normal%2520-
%2520ectocervix%2520%28GTEx%29%2522%252C%2522normal%2520-
%2520endocervix%2520%28GTEx%29%2522%252C%2522normal%2520-
%2520uterus%2520%28GTEx%29%2522%255D%257D&cutoff=%257B%2522value%2522%2
53A30%257D&unit=%2522TPM%2522

Shortened url: https://tinyurl.com/y9fefymf

PCAWG-Scout on demand analysis (1e)

Exclusivity analysis of non-*ERG* fusion donors in PCAWG-Scout

To reproduce the exclusivity analysis, we first need to produce the list of samples without *ERG* fusions. That is done by generating the list of donors with *ERG* fusions and taking them out of the list of all donors with SV (somatic structural variant) data.

To generate the list of ERG fusion donors in Prost-AdenoCa: (1) go to the report for Prost-AdenoCa (http://pcawgscout.bsc.es/entity/Study/Prost-AdenoCa); (2) click on ‘SV summary’ button toward the bottom of the page; (3) in the resulting SV (structural variant) table, select fusions involving *ERG* by filtering the table (click on the ‘filter’ button at the bottom of the table and then, in the popup window, typing ‘*ERG*’ in the field for ‘Gene 1’); (4) after the table is filtered you can find the associated donors by clicking on the ‘column’ button at the bottom of the table and then, in the popup window, clicking ‘save list’ button next to the label 'Fusion donors'. The list will open in a pop-up window. Open the report in the main window using the button with a link-out icon on the popup window header bar. You can rename the list using the ‘Edit’ button on the sidebar of the report. Make it a favorite by clicking on the star icon at the top so it can be used for later analyses.

To find the complement of the list: (1) go back to the report for Prost-AdenoCa (http://pcawgscout.bsc.es/entity/Study/Prost-AdenoCa) and click on the link for 'SV donors'; (2) from the sidebar, select the button 'Compare'; (3) Since the previous list was made a favorite, it now appears in the popup window, where you click the button 'Remove' to generate the complement donor list.

As before you can rename the list and save it as a favorite. For your convenience the list is accessible as **Prost-AdenoCa ERG non-fusion donors** (http://pcawgscout.bsc.es/entity_list/Sample/Prost-AdenoCa ERG non-fusion donors). You can now click on the 'Characteristic alterations' button at the bottom of the page to perform the exclusivity analysis. Doing so will start the on-demand analysis, which should complete in less than a minute. The result is a list of gene alterations that are enriched in the non-fusion donors, with associated statistical significance, as shown in **Figure 1d** (http://pcawgscout.bsc.es/entity_list_action/Sample/characteristic_alterations/Prost-AdenoCa ERG non-fusion donors)

Annotation and 3D clustering of SPOP mutations in PCAWG-Scout

To reproduce the image with the SPOP/PTEN structure, search for SPOP in the search box at the top of the page and select **the first protein isoform** (http://pcawgscout.bsc.es/entity/Protein:Ensembl Protein ID/ENSP00000240327?organism=Hsa/feb2014) from the report sidebar. To show the protein report in the JMOL viewer, click the 'JMOL' tab and select the PDB 4o1v. To display the mutation density gradient overlaid, click the 'PCAWG' tab, then the 'Highlight' button, and go back to the JMOL' tab to show the gradient with respect to all PCAWG donors.

To focus on prostate samples, first click the 'PCAWG' tab, then click on 'filter', type 'Prost-AdenoCa' in the field 'histology_abbreviation', and click 'submit' to filter down to only those samples. Click 'Highlight' and then click on the 'JMOL' tab to view *SPOP* mutations only from prostate samples.

You can use the 'Sequence' tab to visualize the *SPOP* mutation clustering on a linear depiction of protein sequences (not included in figure). For further confirmation click the 'Protein feature incidence' to see a binomial distribution analysis that detects if a region is both significantly mutated and annotated as 'Important for binding substrate proteins'. The mutation 'F -> A' in residue 133 (the most frequent mutation in the cohort) is annotated as 'Strongly reduced affinity for substrate protein'.

Supplementary Note 3. Recreating Figure 5.

1- Select the CNS-tumors from the ‘Meta-cohorts’ tab.

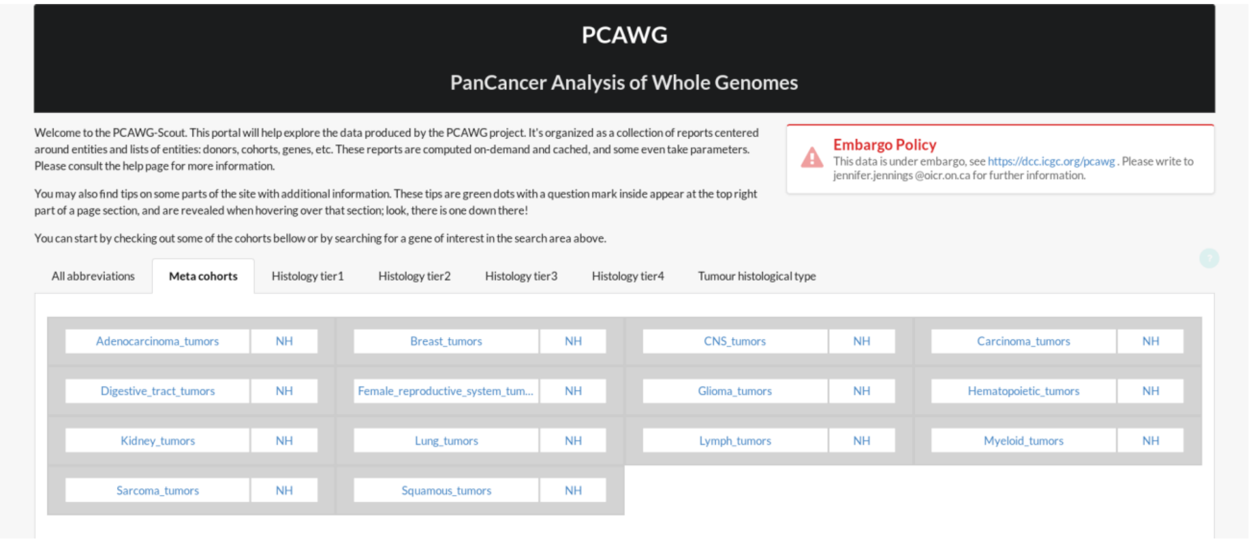

2- On the entity page for the cohort click, on the button for the ‘Gene report’ action and type ‘*IDH1*’.

3- In the gene report, click on the link that points to the 22 samples affected. Doing so will open the sample list report for the samples.

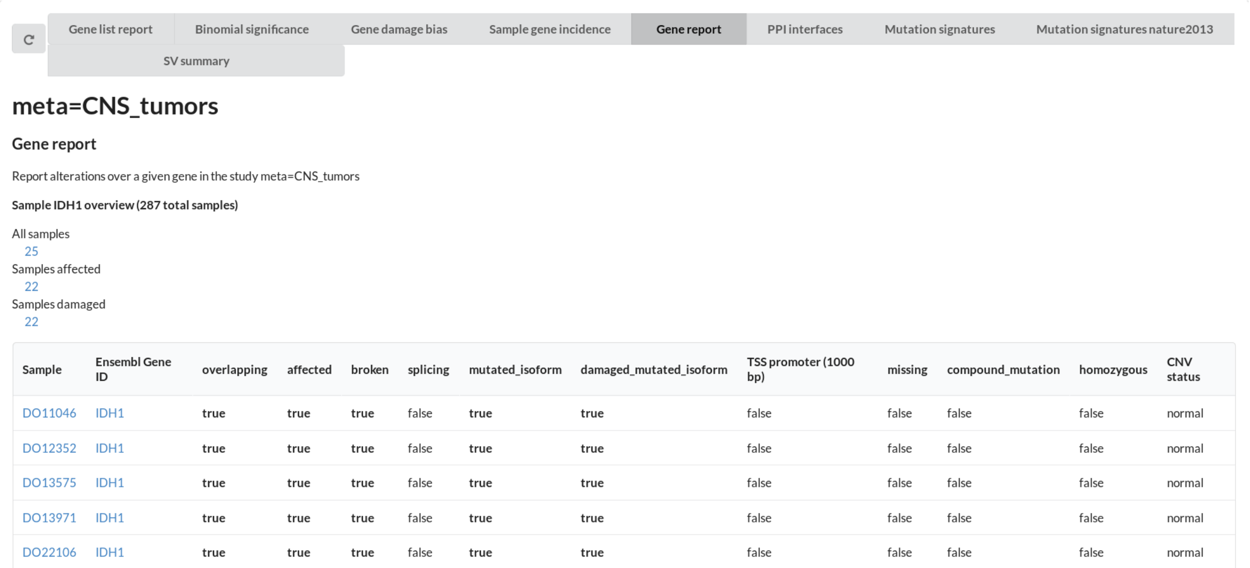

Sample	Ensembl Gene ID	overlapping	affected	broken	splicing	mutated_isoform	damaged_mutated_isoform	TSS promoter (1000 bp)	missing	compound_mutation	homozygous	CNV status
DO11046	IDH1	true	true	true	false	true	true	false	false	false	false	normal
DO12352	IDH1	true	true	true	false	true	true	false	false	false	false	normal
DO13575	IDH1	true	true	true	false	true	true	false	false	false	false	normal
DO13971	IDH1	true	true	true	false	true	true	false	false	false	false	normal
DO22106	IDH1	true	true	true	false	true	true	false	false	false	false	normal

4- Click on the ‘Differential expression’ button and then submit the differential expression analysis. The first table shows all the genes and their corresponding statistics for differential expression between the 22 samples affected by *IDH1* mutations and the rest

of the samples in the cohort. Negative values represent repression.

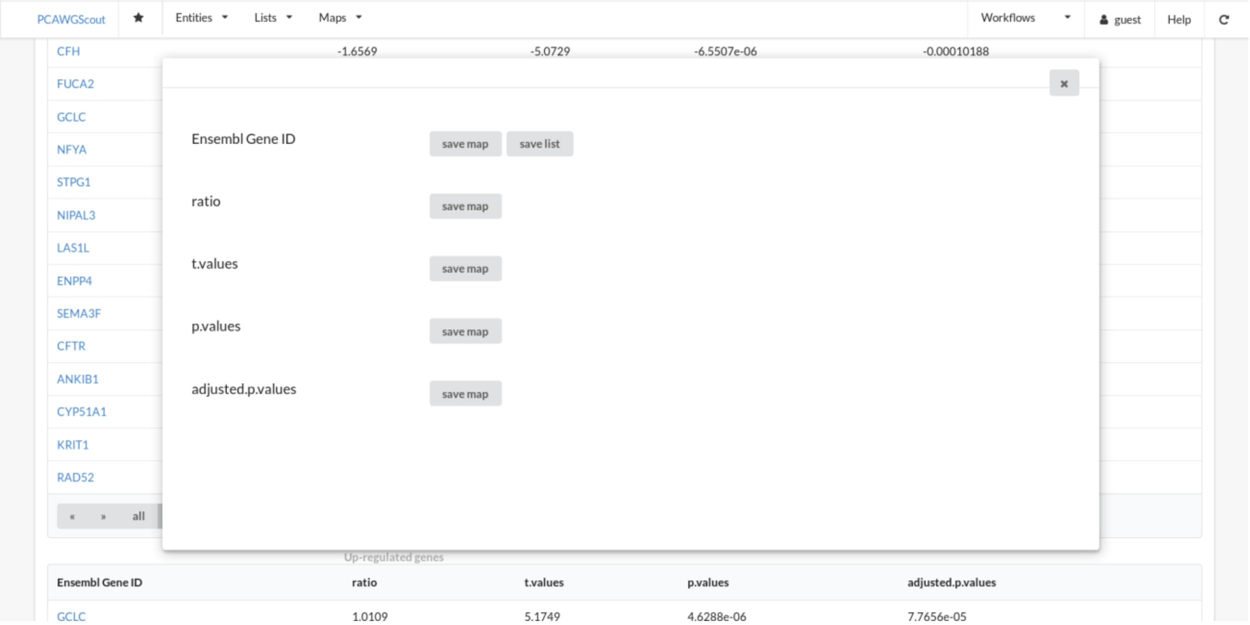

5- Click on the button ‘column’ on the table footer and then on the button ‘Save map’ next to the header ‘t.values’. After a short while, a window will open with a table listing the genes and their corresponding t-values. That is the map for use in coloring the genes. After a few seconds, you will be able to open the map in a different window by clicking on the top right button with a blue link-out icon. From the page accessed, you can rename the map (e.g., as ‘t-values for *IDH1* mutants’). After renaming the map a new page will open. Clicking on the star on the top-bar will turn it yellow. That change of color indicates that the map is placed in your top-bar drawer for favorite maps, under the sub-header ‘Gene’.

6- Go back to the cohort entity report from step 2. Click on the link under the header ‘Genotyped donors’. That will open the sample list report for all cohort donors and feature a table with the clinical information. From that table, use the ‘column’ button to extract the map between samples and the feature ‘donor_survival_time’. As before, open the map into a separate window, rename it, and make sure it is a favorite by clicking on the top-bar star.

7- Go back to the cohort entity report from step 2. Click on the button ‘Gene damage bias’ to open a driver prediction report, which compares the predicted damage of the mutations found in a given protein with the predicted damage of all possible mutations. That highlights proteins harboring mutations significantly more damaging than expected at random. From the table, follow the usual procedure to extract the map between genes and p-values; except this time, before renaming the list, convert p-values to ‘P-value

scores’, which are better suited to be used in plots.

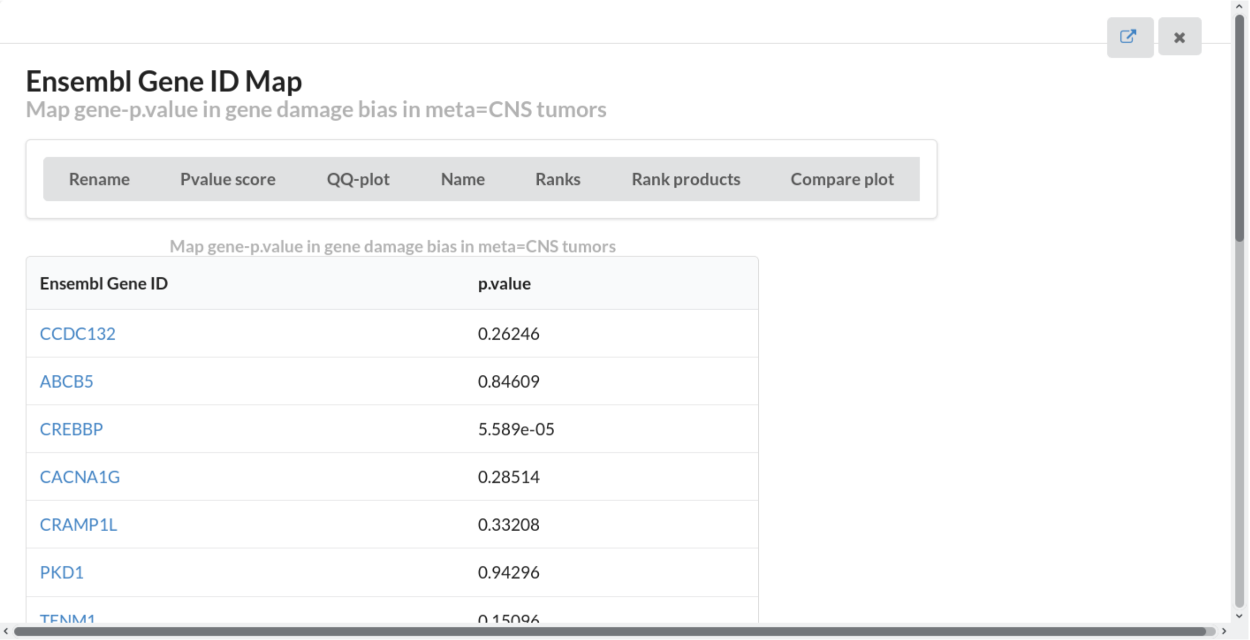

8- We have gathered all of the information needed to annotate the network. The network can be found on the cohort report from step 2. Open the ‘Aesthetics’ menu to map visual characteristics to different values.

9- Select ‘nodes’, the ‘color’ aesthetic, and select the map from step 5. That will color a gradient from the most repressed in purple to the most over-expressed in gold, going through gray, which represents no dysregulation.

10- Perform a similar procedure with the aesthetic ‘borderWidth’ and the survival time map from step 6. That will highlight donors with longer survival times.

11- Repeat the procedure with the aesthetic ‘size’ and the map from step 7 to highlight genes that are significantly damaged.

12- If you click on the edges of the graph, a window will open describing the nature of the relationship between the sample and the gene, e.g. whether the mutation is a validated driver or not. To visualize that information, select the element ‘edges’ and the aesthetic ‘color’, and assign to it the field ‘Validated’. On the map select the option ‘Use textarea’.

In the text area, put the line ‘validated green’ and, below that, the line ‘predicted orange’.

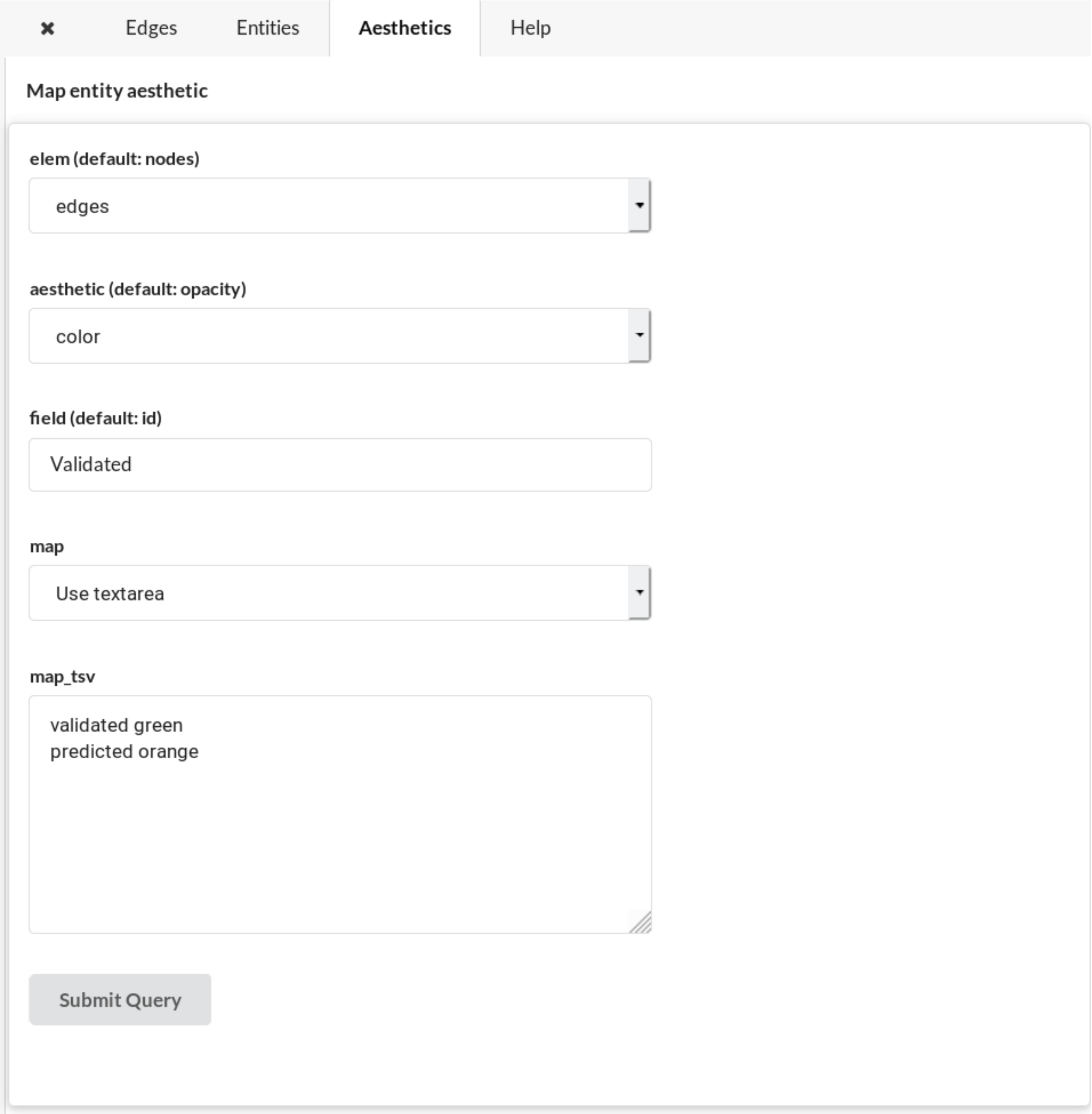

13- You can use the contextual menu to help organize they layout of the nodes, for instance using the ‘surround with first neighbors’ to organize the samples around the main driver genes.

Supplementary Figure 1. PCAWG-scout uses PanDrugs for prediction of recommended therapies.

PanDrugs is a web-based tool (http://www.pandrugs.org) to guide the selection of therapies from the results of genome-wide studies in cancers. It allows identification of actionable molecular alterations and prioritization of drugs by calculating gene-drug scores (GScore and DScore, respectively). Those scores take into account: i) the relevance in cancer of the affected gene and the specific variant type; ii) the target pathway context; iii) the drug approval status (FDA, clinical trial, or experimental small molecule inhibitor); and iv) manually curated pharmacological information retrieved from the literature. PanDrugs GScore indicates the biological relevance in cancer of the gene affected, the functional impact, and the clinical actionability of the specific mutation, integrating evidence from public resources. The DScore indicates the suitability of the drug according to the genomic profile. Together, the two scores combine the biological and clinical relevance of the genes and their susceptibility to be targeted, reflecting the strength of evidence for the gene-drug association. That measure can be used to assist in clinical decision-making. Additionally, it incorporates manually curated information about the drug approval status and its usage in cancer therapies or clinical studies in this field.

The current version of PanDrugs integrates data from 24 sources, supporting more than 56,000 drug-target associations obtained from approximately 4800 genes and 9000 unique compounds.

[1] Piñeiro-Yáñez E. et al. (2018) Genome Medicine. 10:41
[2] Wagner AH. et al. (2016) Nucleic Acids Res. Jan 4;44(D1):D1036-44
[3] Van Allen EM. et al. (2014) Nat Med. Jun;20(6):682-8
[4] Basu A. et al. (2013) Cell. Aug 29;154(5):1151-61
[5] Iorio F. et al. (2016) Cell. Jul 28;166(3):740-54

a) Overview of the drug assignment distribution for 2494 donors for 37 different tumor types. Each bar represents the percentage of patients with a suggested therapy for each tumor type based on simple somatic mutations detected. Different colors correspond to different approval status for the drugs as indicated in the legend. Only high impact alterations (those with a PanDrugs GScore greater or equal to 0.6) are considered. **b)** Example of a therapy suggestion based on evidence from an affected gene. Donor DO13132 with Glioblastoma (CNS-GBM) has an *EGFR* p.Gly719Cys missense mutation, which leads to carcinogenic processes of cell growth and proliferation. The mutation confers sensitivity to *EGFR* inhibitors such as Erlotinib, one of the proposed therapies. **c)** Example of a therapy suggestion against the use of a conventional therapy based on evidence from an affected gene. *EGFR* inhibitor Cetuximab is a standard therapy for the treatment of colorectal cancer, but *KRAS* mutations have shown to be a predictor of resistance to cetuximab therapy. In the DO44094 case, the missense p.Gly12Val mutation indicates resistance to Cetuximab. **d)** Example of an indirect therapy suggestion based on evidence from a pathway membership: Melanoma patient DO220845 has the p.Val600Glu alteration in *BRAF*. The alteration suggests use of *BRAF* inhibitors such as Vemurafenib. It also suggests *MEK* inhibitors, for example Trametinib, according to the downstream position of those gene in relation to *BRAF* in the *MAPK* signaling pathway.

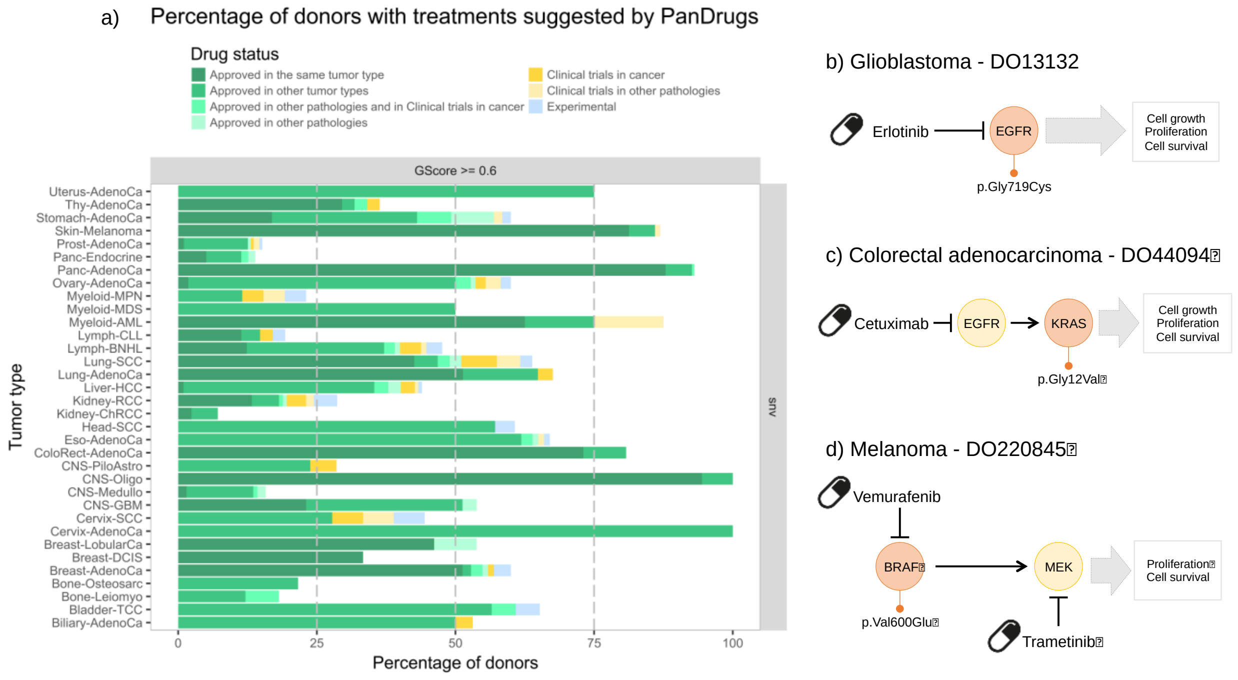
a)
Percentage of donors with treatments suggested by PanDrugs
Drug status
Approved in the same tumor type
Approved in other tumor types
Approved in other pathologies and in Clinical trials in cancer
Approved in other pathologies
Clinical trials in cancer
Clinical trials in other pathologies
Experimental
GScore >= 0.6
snv
Tumor type
Uterus-AdenoCa
Thy-AdenoCa
Stomach-AdenoCa
Skin-Melanoma
Prost-AdenoCa
Panc-Endocrine
Panc-AdenoCa
Ovary-AdenoCa
Myeloid-MPN
Myeloid-MDS
Myeloid-AML
Lymph-CLL
Lymph-BNHL
Lung-SCC
Lung-AdenoCa
Liver-HCC
Kidney-RCC
Kidney-ChRCC
Head-SCC
Eso-AdenoCa
ColoRect-AdenoCa
CNS-PiloAstro
CNS-Oligo
CNS-Medullo
CNS-GBM
Cervix-SCC
Cervix-AdenoCa
Breast-LobularCa
Breast-DCIS
Breast-AdenoCa
Bone-Osteosarc
Bone-Leiomyo
Bladder-TCC
Biliary-AdenoCa
0
25
50
75
100
Percentage of donors
b) Glioblastoma - DO13132
Erlotinib
EGFR
p.Gly719Cys
Cell growth
Proliferation
Cell survival
c) Colorectal adenocarcinoma - DO44094
Cetuximab
EGFR
KRAS
p.Gly12Val
Cell growth
Proliferation
Cell survival
d) Melanoma - DO220845
Vemurafenib
BRAF
p.Val600Glu
MEK
Trametinib
Proliferation
Cell survival

Supplementary Figure 2. View protected data using a local Xena Hub.

The Xena platform has two components: the web-based Xena Browser and the back-end Xena Hubs. The Xena Browser empowers biologists to explore data across multiple Xena Hubs. The back-end Xena Hubs host the genomics data, are configured to be public or private, and can be installed on laptops, public servers, behind a firewall, or in the cloud. Xena Browser simultaneously connects to any number of Xena Hubs, with integration occurring in the browser, enabling data to be distributed across multiple Xena Hubs. To view the controlled-access non-coding simple mutations, download the file that contains those mutations (https://gtrepo-osdc-tcga.annailabs.com/cghub/metadata/analysisFull/86a1f73a-fabf-4abe-a597-a47d84a8c980). Only authorized users can download the protected data. The file is pre-formatted to be imported directly it into a local Xena hub on a user's laptop. Once it is loaded, the UCSC Xena Browser will connect to both the local hub and the public PCAWG hub concurrently, while still keeping the protected data private. The arrangement allows users to visualize the protected whole-genome simple mutation data alongside the open-access PCAWG data. More information about using Xena private data hubs can be found at http://xena.ucsc.edu/private-hubs/ .

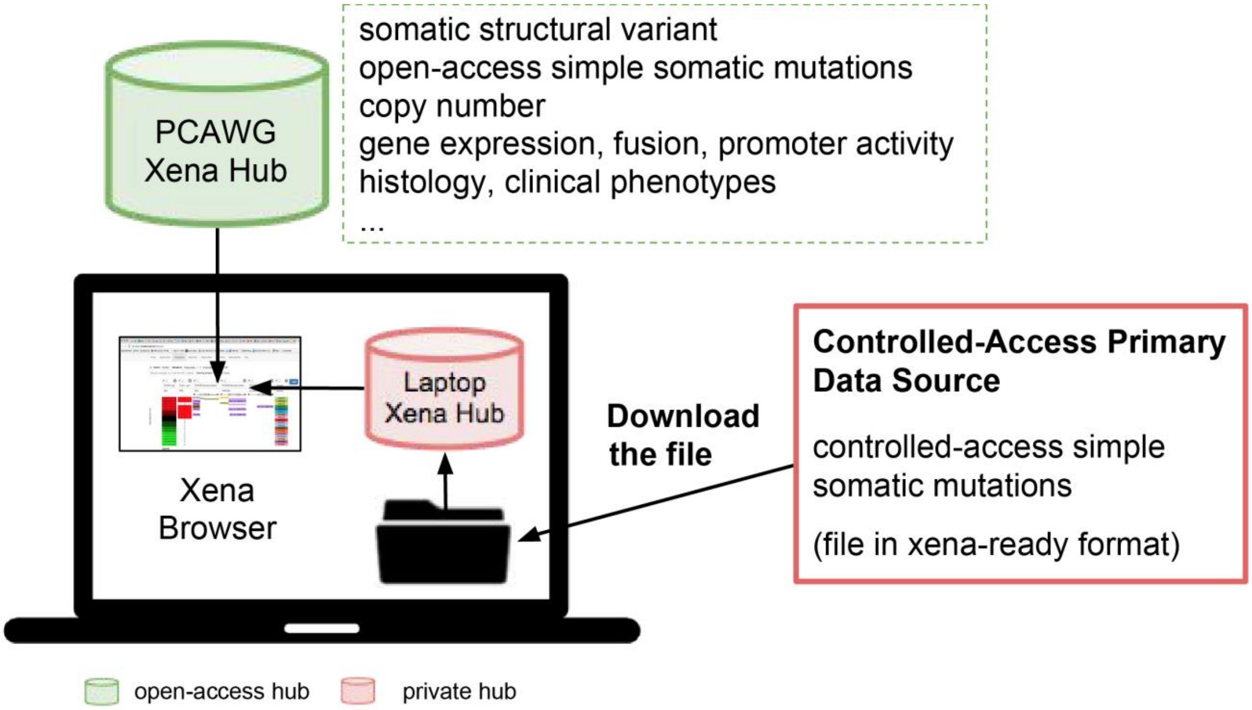

Supplementary Figure 3. Secured PCAWG-Scout installation to support controlled-access data.

To protect controlled access data, in particular genomic mutations, the PCAWG-Scout is configured so that controlled data is on only a siloed machine behind a firewall. Approved analyses that do not compromise the security of the data are made available by configuring a remote workflow access file on the Rbbt installation. Approved analyses can be accessed via a web-browser, javascript plotting utilities, or command-line tools. The PCAWG-Scout machine does not hold the controlled access data, and the silo is not directly accessible from outside, keeping the data secure. Any remote installation of the PCAWG-Scout can request such analyses and will relay them to the silo. The procedure enables the system to be extended by the general research community without requiring all researchers to have access to the controlled data.

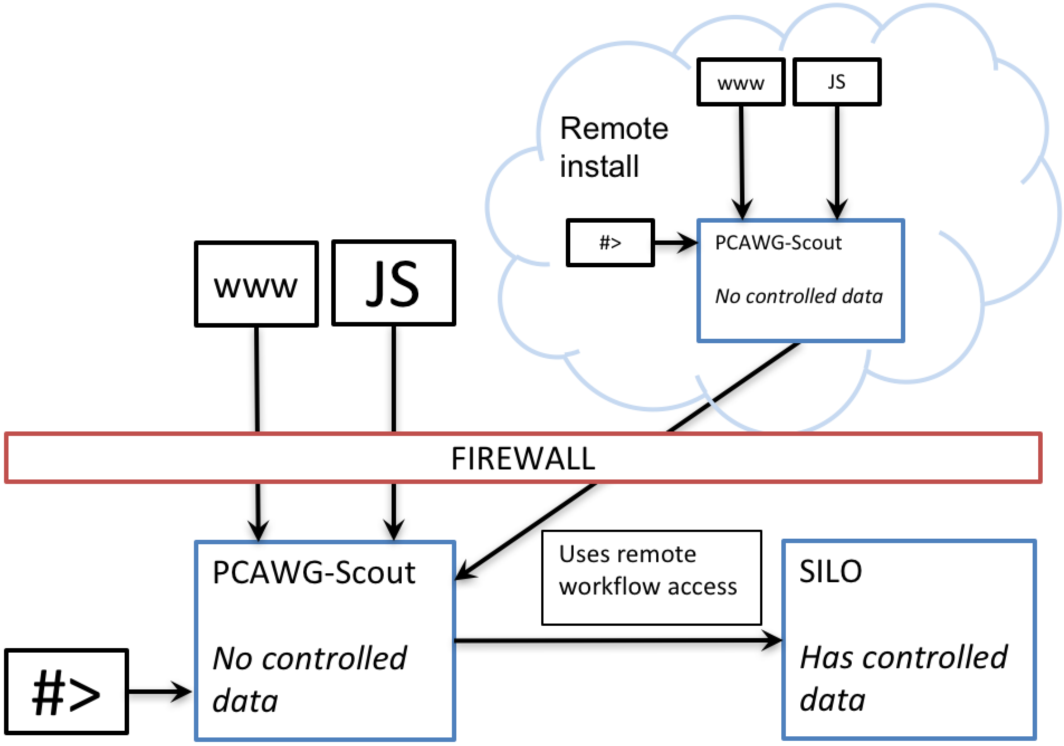

Supplementary Figure 4. Circos plots for the other 7 tumors with ERG fusion for all chromosomes (left side of figures) and for chromosome 21 (right side of figures).

Donor PRAD-US::59207bcd-f127-481f-b576-8716da7d7d97 (DO36372)

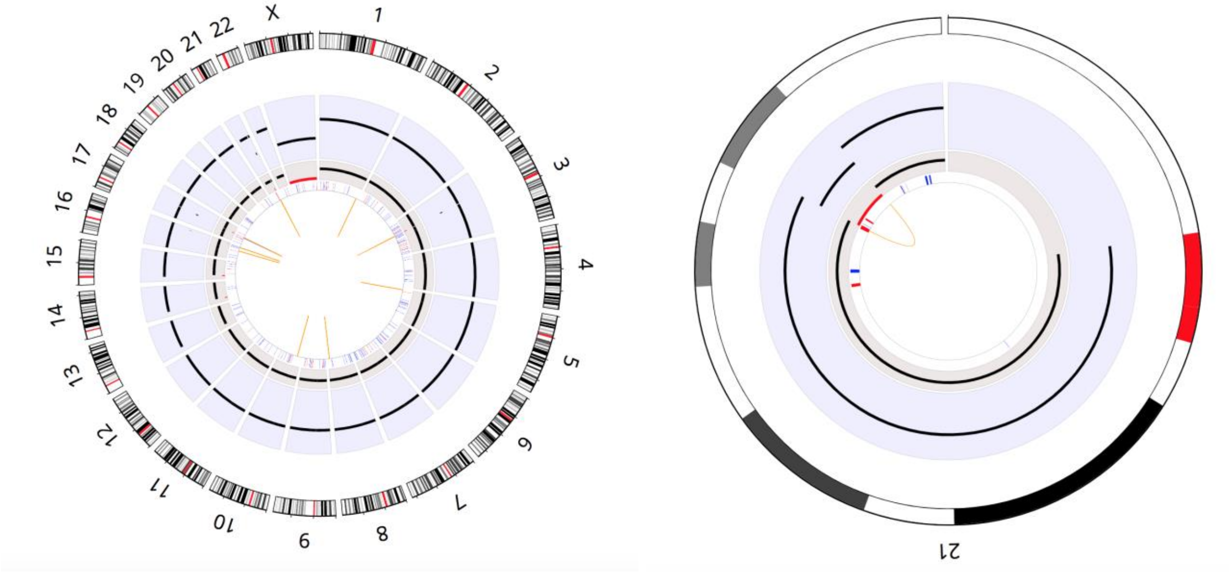

Donor PRAD-US::6845076e-e940-4437-8618-81db4a447544 (DO36345)

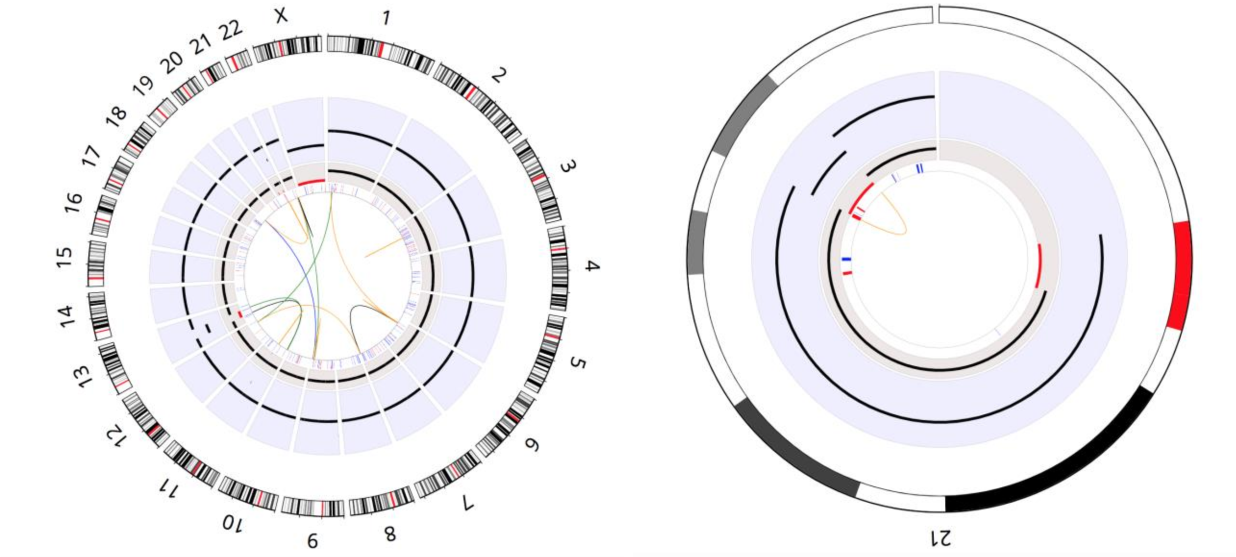

Donor PRAD-US::87959988-bb25-47ee-afc1-9af92592bbe7 (DO36273)

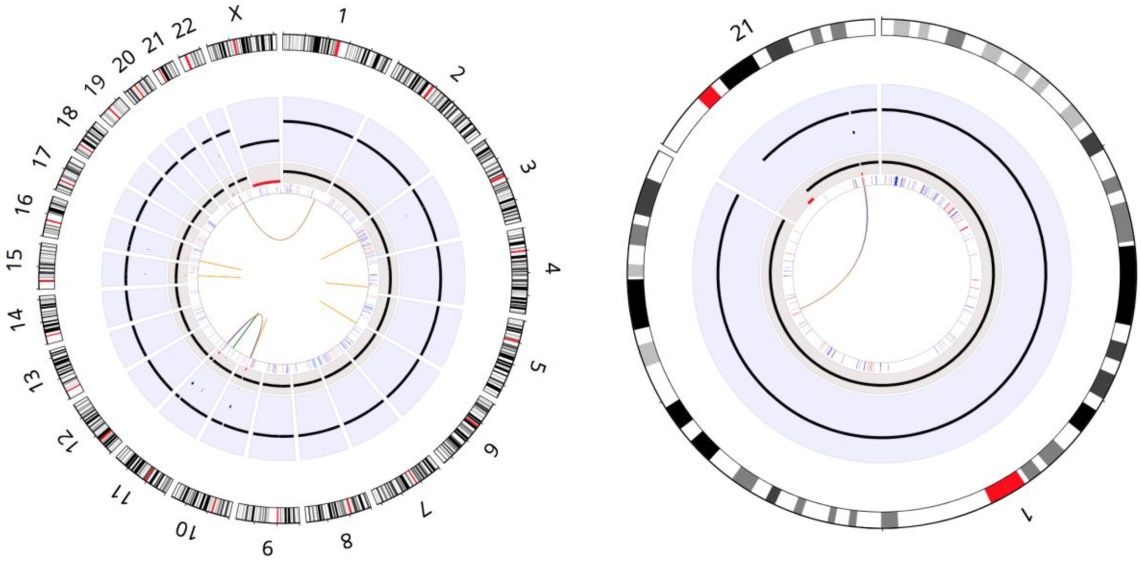

Donor PRAD-US::aa252f78-ebf3-42f9-86af-bb6e57345706 (DO36359)

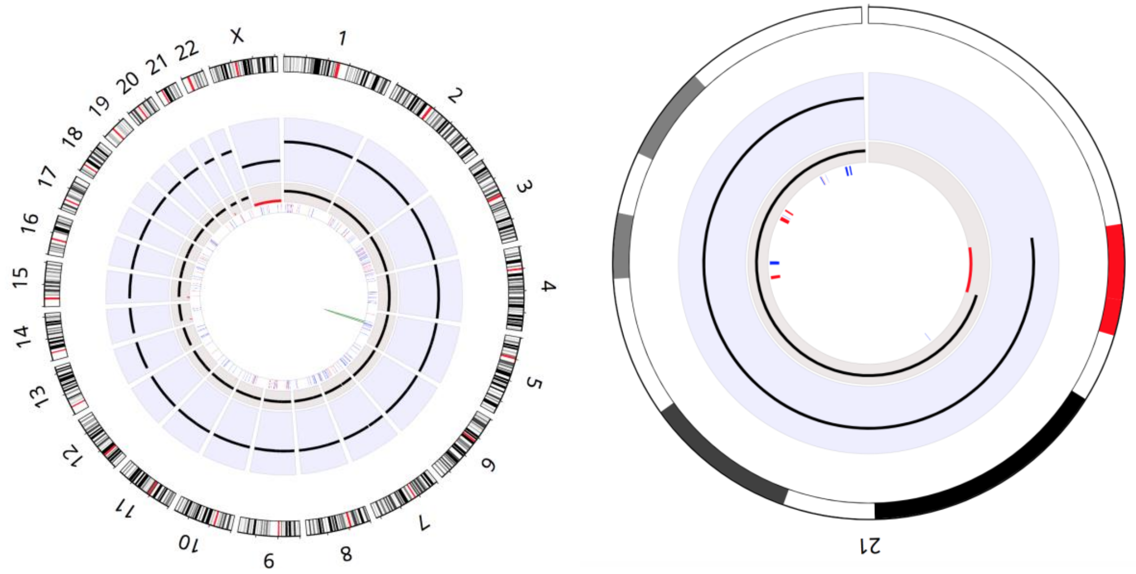

Donor PRAD-US::be5411dc-3caa-4b25-9b94-ce463e8e52b4 (DO36265)

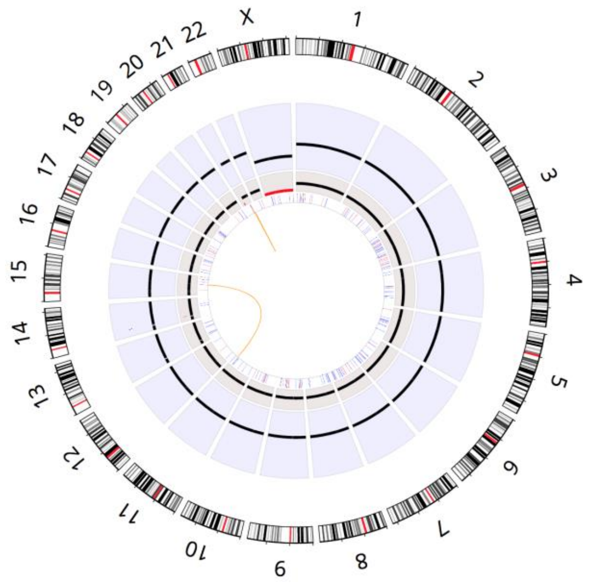

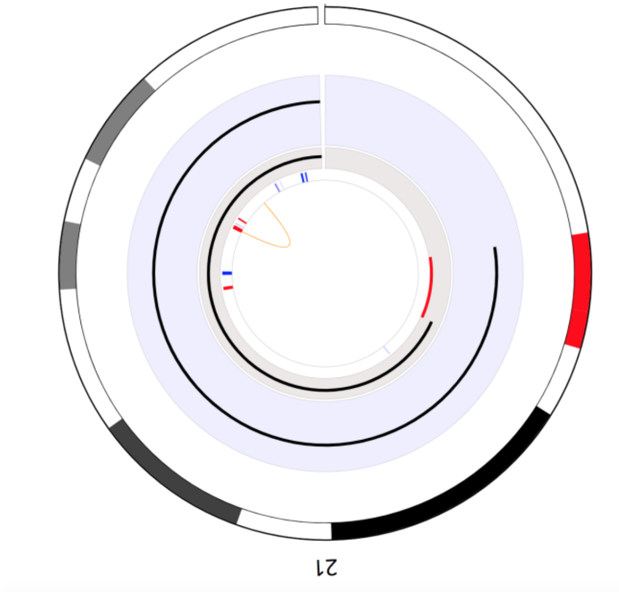

Donor PRAD-US::e5e4d3b6-8e58-4dcf-8f67-a00e656a8e27 (DO36335)

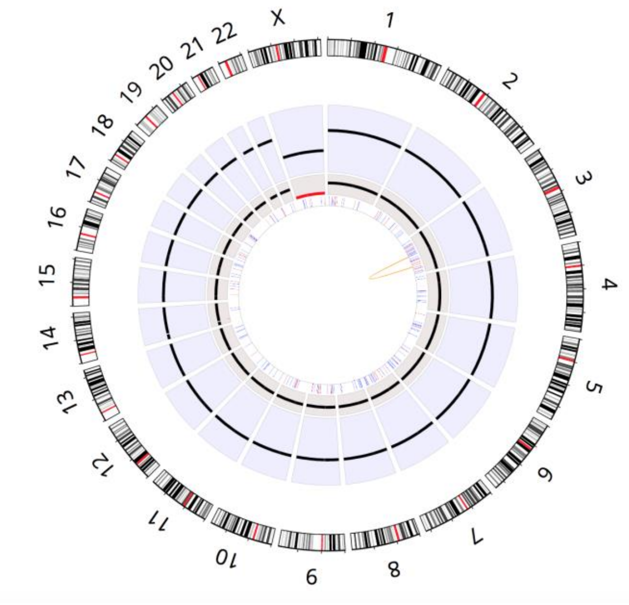

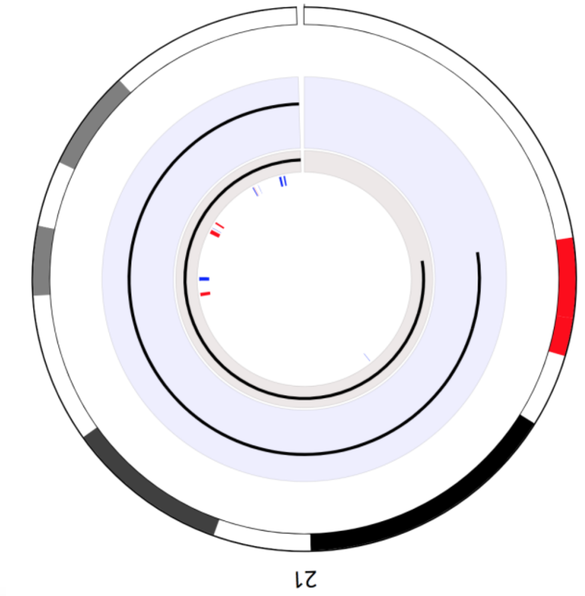

Donor PRAD-US::f6381367-142c-45d0-92b3-c1727d1813ce (DO36283)

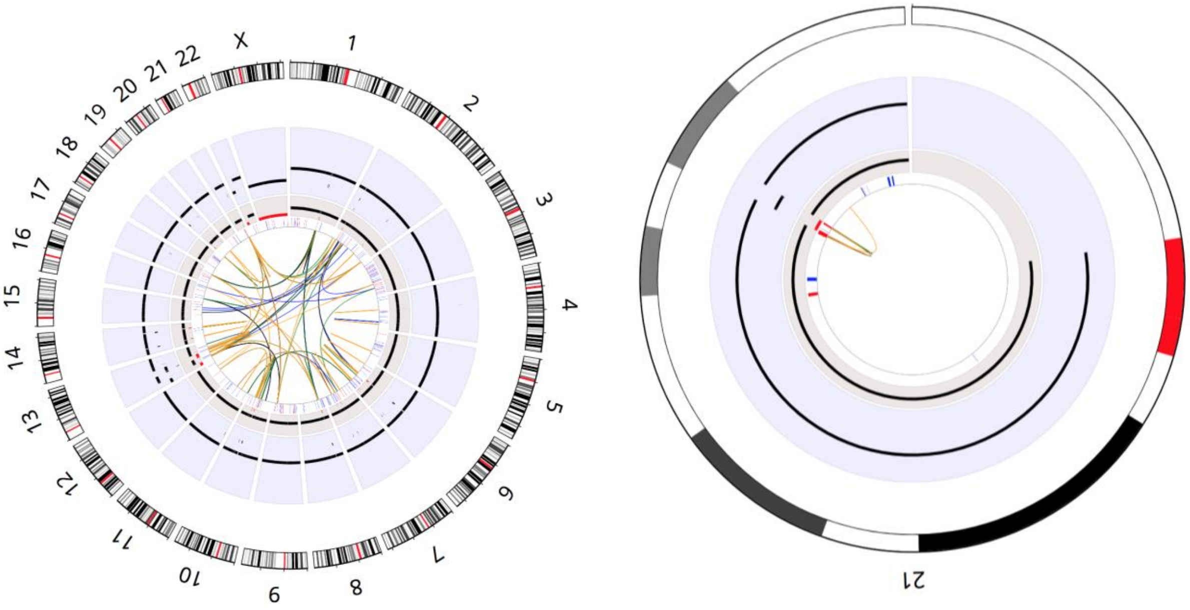

Supplementary Figure 5. Prostate adenocarcinoma *ERG* structural variants

A UCSC Xena Visual Spreadsheet showing that 106 of the 195 prostate adenocarcinoma tumors showed the *ERG* fusion that joins an external piece of DNA from the 5' direction, replacing the 5' end of *ERG* with another piece of DNA (promoter fusion). The 195 prostate tumors were from four different projects: PRAD-CA, PRAD-US, PRAD-UK and EOPC-DE. Eighty-nine of the 195 prostate tumors (46%) did not show that type of fusion . Structural variants in *ERG* were detected in three samples, but they were not the 5' joining type (DO52498, DO50430, and DO51087). For structural variants (column B), the grey-colored lines represent the external DNA segments found to be fused to *ERG*, and the short black tick marks indicate the breakpoints in *ERG*. The breakpoints are clustered at the beginning of the coding regions. A live view can be accessed here: https://xenabrowser.net/heatmap/?bookmark=4d07f983ccd528022a931703aa1abe47.

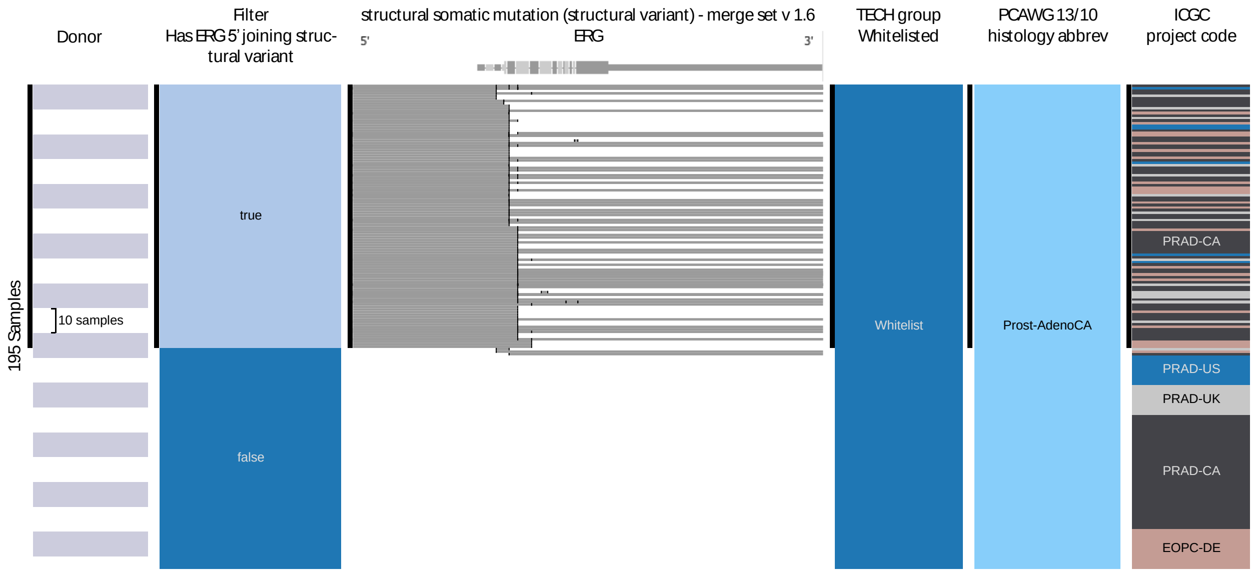

Supplementary Table 1. Code availability.

Resource	Open source code availability
ICGC Data Portal	https://github.com/icgc-dcc/dcc-portal
PCAWG-Scout	http://mikisvaz.github.io/rbbt/; https://github.com/Rbbt-Workflows; https://github.com/Rbbt-Apps/PCAWGScout
UCSC Xena Browser	https://github.com/ucscXena/ucsc-xena-client
Expression Atlas	https://github.com/gxa/atlas
Chromothripsis Explorer	https://github.com/parklab/ShatterSeek

Supplementary Table 2. Embeddable javascript modules.

Javascript Module	Utility	Open source code availability
OncoGrid	Generate OncoGrids and related tracks	https://github.com/oncojs/oncogrid
Xena Visual Spreadsheet	Generate visual spreadsheet	https://github.com/ucscXena/ucsc-xena-client
Kaplan-Meier	Kaplan-meier estimator and log-rank test	https://github.com/ucscXena/kaplan-meier
static-interval-tree	Fast overlapping interval queries in javascript	https://github.com/ucscXena/static-interval-tree
Expression Atlas Widget: Heatmap & Anatomogram	View tissue-specific results on a heatmap and human figure	https://github.com/gxa/atlas-heatmap

Supplementary Table 3. List of PCAWG primary results supported by online visualization resources.

Table of primary results generated by PCAWG analysis working groups available for visualization by UCSC Xena, Expression Atlas, PCAWG-Scout, and Chromothripsis Explorer. Each primary result is referenced by corresponding synapse IDs. Synapse folder ID is the identifier for the synapse landing page for each type of primary results. The landing page typically includes a summary written by the analysis working group to briefly describe the bioinformatics methods used and a list of results generated. Because there are often multiple versions of the same results files (such as fpkm vs fpkm-uq gene expression estimations, or simple mutations from all specimens or aggregated by donors), synapse identifiers in the remaining columns point to the actual data file ingested by each online resource. The data snapshot was taken as of Feb 10, 2017.

Data	Synapse page ID	UCSC Xena	Expression Atlas	PCAWG-Scout	Chromo-thripsis Explorer
Consensus SNVs and indels	syn7118450	syn7364923<br>syn7364924		syn7364923	syn7357330
Consensus SVs	syn5964535	syn7596712		syn7596712	syn7596712
Consensus copy number	syn8042880	syn8042988		syn8042992	syn8042880
Gene expression	syn3104297	syn5553991	syn5553983<br>syn5553985	syn5553991	
GTEx gene expression derived using the PCAWG RNA-seq SOP	syn8105922		syn8105922		
RNAseq gene fusion	syn7221157	syn7221157			

RNAseq alternative promoter usage	syn3354819	syn10332949			
small RNA-Seq (miRNA) analyses	syn5842981	syn5878064<br>syn5878067			
Patient-centric driver catalogue	syn7250534	syn11050201		syn7328242	
Integrated driver calls	syn7359546			syn8035740	
APOBEC mutagenesis analysis	syn7437205	syn7511424			
Tumour subtype and histology information	syn4974831	syn10389164	syn7253569	syn7253569	syn4974831 (version 9)
Donor clinical data	syn4974831	syn10389158		syn7772065	syn4974831 (version 9)
Consensus purity and ploidy					syn8272483
