# Supplementary material for: A user guide for the online exploration and visualization of PCAWG data
Source: Nat Commun. 2020 Jul 7;11:3400. doi: 10.1038/s41467-020-16785-6 (PMC7340791; doi:10.1038/s41467-020-16785-6)
Supplement: Supplementary file 2 — Reporting Summary [file 41467_2020_16785_MOESM2_ESM.pdf]

nature research

Corresponding author(s): Mary Goldman, mary@soe.ucsc.edu

Last updated by author(s): Apr 11, 2020

Reporting Summary

Nature Research wishes to improve the reproducibility of the work that we publish. This form provides structure for consistency and transparency in reporting. For further information on Nature Research policies, see Authors & Referees and the Editorial Policy Checklist .

Statistics

For all statistical analyses, confirm that the following items are present in the figure legend, table legend, main text, or Methods section.

n/a	Confirmed	
☒	☐	The exact sample size (*n*) for each experimental group/condition, given as a discrete number and unit of measurement
☒	☐	A statement on whether measurements were taken from distinct samples or whether the same sample was measured repeatedly
☒	☐	The statistical test(s) used AND whether they are one- or two-sided<br>*Only common tests should be described solely by name; describe more complex techniques in the Methods section.*
☒	☐	A description of all covariates tested
☒	☐	A description of any assumptions or corrections, such as tests of normality and adjustment for multiple comparisons
☒	☐	A full description of the statistical parameters including central tendency (e.g. means) or other basic estimates (e.g. regression coefficient) AND variation (e.g. standard deviation) or associated estimates of uncertainty (e.g. confidence intervals)
☒	☐	For null hypothesis testing, the test statistic (e.g. *F*, *t*, *r*) with confidence intervals, effect sizes, degrees of freedom and *P* value noted<br>*Give P values as exact values whenever suitable.*
☒	☐	For Bayesian analysis, information on the choice of priors and Markov chain Monte Carlo settings
☒	☐	For hierarchical and complex designs, identification of the appropriate level for tests and full reporting of outcomes
☒	☐	Estimates of effect sizes (e.g. Cohen's *d*, Pearson's *r*), indicating how they were calculated

Our web collection on statistics for biologists contains articles on many of the points above.

Software and code

Policy information about availability of computer code

Data collection	N/A - All data was provided by the PCAWG consortium. No data was collected as part of this paper.
Data analysis	The core computational pipelines used by the PCAWG Consortium for alignment, quality control and variant calling are available to the public at https://dockstore.org/search?search=pcawg under the GNU General Public License v3.0, which allows for reuse and distribution. The code for all tools in this paper are open source and publicly available. Code for the ICGC Data Portal is available at https://github.com/icgc-dcc/dcc-portal. Code for the UCSC Xena Browser is available at https://github.com/ucscXena/ucsc-xena-client. Code for the Chromothripsis Explorer is available at https://github.com/parklab/ShatterSeek. Code for the Expression Atlas is at https://github.com/gxa/atlas. Code for PCAWG-Scout is at http://mikisvaz.github.io/rbbt/, https://github.com/Rbbt-Workflows, and https://github.com/Rbbt-Apps/PCAWGScout.

For manuscripts utilizing custom algorithms or software that are central to the research but not yet described in published literature, software must be made available to editors/reviewers. We strongly encourage code deposition in a community repository (e.g. GitHub). See the Nature Research guidelines for submitting code & software for further information.

Data

Policy information about availability of data

All manuscripts must include a data availability statement. This statement should provide the following information, where applicable:

- Accession codes, unique identifiers, or web links for publicly available datasets
- A list of figures that have associated raw data
- A description of any restrictions on data availability

Somatic and germline variant calls, mutational signatures, subclonal reconstructions, transcript abundance, splice calls and other core data generated by the ICGC/TCGA Pan-cancer Analysis of Whole Genomes Consortium is described here1 and available for download at https://dcc.icgc.org/releases/PCAWG. Additional information on accessing the data, including raw read files, can be found at https://docs.icgc.org/pcawg/data/. In accordance with the data access policies of the ICGC and TCGA projects, most molecular, clinical and specimen data are in an open tier which does not require access approval. To access potentially identification

information, such as germline alleles and underlying sequencing data, researchers will need to apply to the TCGA Data Access Committee (DAC) via dbGaP (https://dbgap.ncbi.nlm.nih.gov/aa/wga.cgi?page=login) for access to the TCGA portion of the dataset, and to the ICGC Data Access Compliance Office (DACO; http://icgc.org/daco) for the ICGC portion. In addition, to access somatic single nucleotide variants derived from TCGA donors, researchers will also need to obtain dbGaP authorization.

Derived data sets described specifically in this manuscript can be found at these locations:

UCSC Xena
https://www.synapse.org/#!Synapse:syn7364923 and https://www.synapse.org/#!Synapse: syn7364924 for consensus SNVs and indels.
https://www.synapse.org/#!Synapse:syn7596712 for consensus SVs.
https://www.synapse.org/#!Synapse:syn8042988 for consensus copy number.
https://www.synapse.org/#!Synapse:syn5553991 for gene expression.
https://www.synapse.org/#!Synapse:syn7221157 for RNAseq gene fusion.
https://www.synapse.org/#!Synapse:syn10332949 for RNAseq alternative promoter usage.
https://www.synapse.org/#!Synapse:syn5878064 and https://www.synapse.org/#!Synapse:syn5878067 for small RNA-Seq (miRNA) analyses.
https://www.synapse.org/#!Synapse:syn11050201 for patient-centric driver catalogue.
https://www.synapse.org/#!Synapse:syn7511424 for APOBEC mutagenesis analysis.
https://www.synapse.org/#!Synapse:syn10389164 for tumour subtype and histology information.
https://www.synapse.org/#!Synapse:syn10389158 for donor clinical data.

Chromothripsis Explorer
https://www.synapse.org/#!Synapse:syn7357330 for consensus SNVs and indels.
https://www.synapse.org/#!Synapse:syn7596712 for consensus SVs.
https://www.synapse.org/#!Synapse:syn8042880 for consensus copy number.
https://www.synapse.org/#!Synapse:syn4974831 version 9 for tumour subtype and histology information, and donor clinical data.
https://www.synapse.org/#!Synapse:syn8272483 for consensus purity and ploidy.

Expression Atlas
https://www.synapse.org/#!Synapse:syn5553983 and https://www.synapse.org/#!Synapse:syn5553985 for PCAWG gene expression.
https://www.synapse.org/#!Synapse:syn8105922 for GTEx gene expression derived using the PCAWG RNA-seq SOP.
https://www.synapse.org/#!Synapse:syn7253569 for tumour subtype and histology information.

PCAWG-Scout
https://www.synapse.org/#!Synapse:syn7364923 consensus SNVs and indels.
https://www.synapse.org/#!Synapse:syn7596712 for consensus SVs.
https://www.synapse.org/#!Synapse:syn8042992 for consensus copy number.
https://www.synapse.org/#!Synapse:syn5553991 for gene expression.
https://www.synapse.org/#!Synapse:syn7328242 for patient-centric driver catalogue.
https://www.synapse.org/#!Synapse:syn8035740 for integrated driver calls.
https://www.synapse.org/#!Synapse:syn7253569 for tumour subtype and histology information.
https://www.synapse.org/#!Synapse:syn7772065 for donor clinical data.

The source data underlying Figures 1-5 are provided as a Source Data file.

Field-specific reporting

Please select the one below that is the best fit for your research. If you are not sure, read the appropriate sections before making your selection.

☒ Life sciences ☐ Behavioural & social sciences ☐ Ecological, evolutionary & environmental sciences

For a reference copy of the document with all sections, see nature.com/documents/nr-reporting-summary-flat.pdf

Life sciences study design

All studies must disclose on these points even when the disclosure is negative.

Sample size	N/A - All data was provided by the PCAWG consortium. No data was collected as part of this paper.
Data exclusions	N/A - All data was provided by the PCAWG consortium. No data was collected as part of this paper.
Replication	N/A - All data was provided by the PCAWG consortium. No data was collected as part of this paper.
Randomization	N/A - All data was provided by the PCAWG consortium. No data was collected as part of this paper.
Blinding	N/A - All data was provided by the PCAWG consortium. No data was collected as part of this paper.

Reporting for specific materials, systems and methods

We require information from authors about some types of materials, experimental systems and methods used in many studies. Here, indicate whether each material, system or method listed is relevant to your study. If you are not sure if a list item applies to your research, read the appropriate section before selecting a response.

Materials & experimental systems

n/a	Involved in the study
☒	☐ Antibodies
☒	☐ Eukaryotic cell lines
☒	☐ Palaeontology
☒	☐ Animals and other organisms
☒	☐ Human research participants
☒	☐ Clinical data

Methods

n/a	Involved in the study
☒	☐ ChIP-seq
☒	☐ Flow cytometry
☒	☐ MRI-based neuroimaging
